# Estrogens determine the efficacy of cancer immunotherapy in obese males with melanoma

**DOI:** 10.1172/jci.insight.189758

**Published:** 2025-06-12

**Authors:** Eloïse Dupuychaffray, Hélène Poinot, Aurélie Vuilleumier, Maxime Borgeaud, Montserrat Alvarez, Betül Taskoparan, Olivier Preynat-Seauve, Clarissa D. Voegel, Eliana Marinari, Denis Migliorini, Valérie Dutoit, Carole Bourquin, Aurélien Pommier

**Affiliations:** 1 School of Pharmaceutical Sciences, and; 2 Institute of Pharmaceutical Sciences of Western Switzerland, University of Geneva, Geneva, Switzerland.; 3 Department of Oncology, Geneva University Hospitals (HUG), Geneva, Switzerland.; 4 Department of Pathology and Immunology, Faculty of Medicine, University of Geneva, Geneva, Switzerland.; 5 Deparment of Nephrology and Hypertension, Inselspital, Bern University Hospital, University of Bern, Bern, Switzerland.; 6 Center for Translational Research in Onco-Hematology, University of Geneva, Geneva, Switzerland.; 7 Agora Cancer Research Center, Lausanne, Switzerland.; 8 Swiss Cancer Center Léman (SCCL), Lausanne and Geneva, Switzerland.; 9 Department of Anaesthetics, Pharmacology, Intensive Care and Emergencies, Faculty of Medicine, University of Geneva, Geneva, Switzerland.; 10 Institute of Pharmacology, Faculty of Medicine, University of Bern, Bern, Switzerland.

**Keywords:** Immunology, Oncology, Cancer immunotherapy, Obesity

## Abstract

Although obesity is a major risk factor for cancer, it may also improve the response to cancer therapy. Here we investigated the impact of obesity on the efficacy of immune checkpoint inhibitors (ICI). In male mice, obesity promoted tumor growth but enhanced the response to ICI. This was associated with higher expression of immune-related genes within the tumor and enhanced infiltration of tumor-specific CD8^+^ T cells. Further, obesity in mice was associated with higher estrogen levels and enrichment of estrogen response genes in the tumor, and anti–programmed cell death 1 (anti–PD-1) efficacy was reduced upon administration of the aromatase inhibitor letrozole, which blocks the production of estrogens. Mechanistically, adipocyte-derived estrogens increased antigen presentation by dendritic cells and tumor-specific CD8^+^ T cell cytotoxicity. Last, overweight and obese men with melanoma responded better to ICI, with high estrogen levels being associated with improved response and survival. Our results suggest that estrogens may serve as a predictive factor of response to ICI in men with melanoma.

## Abstract 2

<span>We show that blood estrogen levels may serve as a predictive factor of response to immunotherapy in owerweight and obese men with melanoma.</span>

## Introduction

Recent advances in cancer immunotherapy have considerably improved clinical response in multiple cancer types (1). Immune checkpoint inhibitors (ICI) targeting programmed cell death 1 (PD-1) or cytotoxic T lymphocyte-associated protein 4 (CTLA-4) are standard of care for advanced-stage melanoma and have considerably improved patient survival (2). Unfortunately, only a minority of patients respond to ICI, with an overall response rate of 30%–50% (3). Resistance to ICI is multifactorial and involves loss of antigenicity, defects in antigen presentation, tumor-mediated immunosuppression and exclusion, and expression of other immune checkpoint molecules (4). Interestingly, although obesity is associated with increased cancer incidence, some studies suggest that ICI efficacy may be increased in obese patients with cancer (5–7). Notably, Cortellini et al. observed that obese patients showed improved responses to ICI across multiple cancer types, in particular melanoma, and they suggested using body mass index (BMI) as a predictive factor of response (8). Interestingly, McQuade et al. found that obesity had a sex-specific effect on response to ICI in patients with melanoma, where high BMI was associated with longer survival in men but not in women (5). These studies suggest that obesity is a relevant yet underexplored factor that affects efficacy of ICI.

Mechanistically, Wang et al. have shown that the upregulation of leptin in obese patients can enhance the expression of PD-1 on T cells, leading to an improved response to PD-1 blockade in obese people (9). Another study demonstrated that leptin promoted the repolarization of tumor-associated macrophages into pro-inflammatory M1 macrophages, enhancing efficacy of ICI (10). Obesity is associated with various physiopathological disorders, such as type 2 diabetes, cardiovascular diseases, and hormonal dysregulation (11), suggesting that other mechanisms may also be involved in obesity-mediated ICI sensitivity.

Together, these data led us to investigate other possible mechanisms behind the interplay between obesity, sex, and response to ICI. In melanoma-bearing mice, we showed that PD-1 blockade is efficient in obese but not in nonobese males, and we identified estrogen signaling as a pathway involved in this obesity-associated antitumor immune response. Indeed, the high expression of the aromatase enzyme in the adipose tissue increases the conversion of testosterone into estrogens, thus defining adipose tissue as a major production site of estrogens in obese males (12). Mechanistically, we demonstrated that adipocyte-derived estrogens enhanced priming of cytotoxic T cells by dendritic cells and increased antigen-specific T cell–mediated cytotoxicity. Finally, we investigated the translational aspect of our findings in patients with melanoma receiving ICI and observed a positive association between estrogen levels and clinical outcomes in men. This work may help improve the stratification of patients with cancer eligible for ICI and contribute to the development of therapeutic strategies combining ICI and estrogen-based therapies.

## Results

### Obesity confers sensitivity to anti–PD-1 treatment in B16-F10 melanoma tumors.

**Figure 1:**
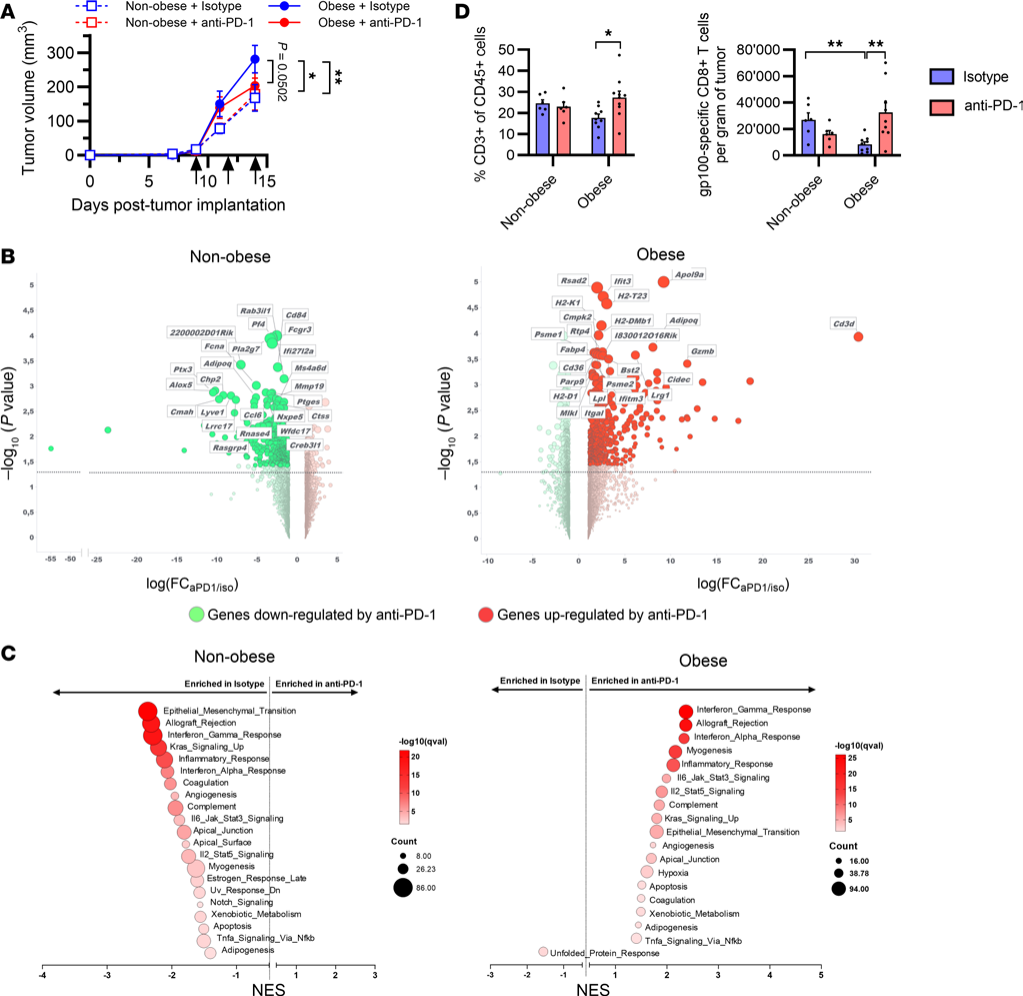
Obesity confers sensitivity to anti–PD-1 treatment in mice bearing B16-F10 tumors. Male C57BL/6 mice fed with a Western diet to induce obesity or with a control diet were subcutaneously injected with B16-F10 tumor cells. After the development of palpable tumors, mice received either anti–PD-1 or isotype control. (**A**) Tumor growth in nonobese (dotted line) or obese mice (solid line), receiving anti–PD-1 (red) or isotype control (blue). Black arrows indicate anti–PD-1 or isotype treatment (*n* = 6–10/group). Two-way ANOVA with Tukey’s post hoc test was used to assess statistical significance. *P < 0.05, **P < 0.01. (**B**) Volcano plots of RNA-sequencing data from B16-F10 tumors from nonobese males (left) and obese males (right) (*n* = 3/group). Red circles and green circles represent differentially expressed genes upregulated or downregulated, respectively, following anti–PD-1 treatment. The 25 most significantly differentially expressed genes are labeled. (**C**) Gene set enrichment analysis (GSEA) of tumors from nonobese mice (left) and obese mice (right) showing all the gene sets significantly enriched in isotype- or anti–PD-1–treated mice (*n* = 3/group). (**D**) Tumor infiltration of CD3^+^ T cells and gp100-specific CD8^+^ T cells, measured by flow cytometry (*n* = 6–10/group). Unpaired 2-tailed Student’s *t* test was used. *P < 0.05, **P < 0.01. NES, normalized enrichment score.

To investigate how obesity affects the response to ICI in melanoma, obese and nonobese male mice were subcutaneously injected with B16-F10 melanoma cells and treated with anti–PD-1 or isotype control antibodies after development of palpable tumors. Obese mice developed significantly larger tumors than nonobese mice (Figure 1A), verifying the pro-tumoral effect of obesity previously described (9, 13, 14). In nonobese mice, no difference in tumor growth was observed between mice receiving anti–PD-1 or isotype control antibodies. In contrast, tumor growth in obese mice was delayed by anti–PD-1, suggesting that obese mice were sensitive to the treatment, though the difference was not significant in this experiment (*P* = 0.0502). Transcriptomic analysis of bulk tumors revealed a distinct effect of PD-1 blockade in nonobese versus obese mice (Figure 1, B and C). In nonobese mice, only 15 genes were upregulated in response to anti–PD-1, whereas in obese mice anti–PD-1 treatment led to upregulation of 279 genes. These differentially expressed genes were mainly involved in immune-related processes, such as response to IFN-γ or antigen processing and presentation (Supplemental Figure 1A; supplemental material available online with this article; https://doi.org/10.1172/jci.insight.189758DS1). Quantitative PCR (qPCR) analysis of some immune-related differentially expressed genes supported the activation of a T cell–mediated immune response following anti–PD-1 treatment in obese mice only (Supplemental Figure 1B). Interestingly, gene set enrichment analysis (GSEA) revealed that anti–PD-1 treatment in obese mice led to enrichment of gene sets that were in contrast reduced in response to anti–PD-1 in nonobese mice (Figure 1C). These analyses highlighted that PD-1 blockade had distinct effects on the transcriptome within the tumors of obese versus nonobese mice. Furthermore, immunophenotyping of bulk tumors revealed that anti–PD-1 treatment in obese mice was associated with an increase in the percentage of infiltrating CD3^+^ T cells, along with a higher number of effector CD8^+^ T cells expressing CD69 and CD44 activation markers (Figure 1D and Supplemental Figure 2C). Further, infiltration of tumor antigen-specific CD8^+^ T cells was reduced in tumors of untreated obese mice compared with nonobese mice (Figure 1D). Anti–PD-1 treatment led to a 4-fold increase in tumor antigen-specific CD8^+^ T cells in obese mice but not in nonobese mice. Together, these results showed that, in mice, obesity is associated with greater T cell–mediated antitumor immune responses to PD-1 blockade.

As B16-F10 tumors are poorly immunogenic and show poor response to PD-1 blockade (15), we investigated the impact of obesity on response to ICI in murine MC38 colorectal tumors, which are immunogenic and sensitive to anti–PD-1 (14). In contrast with B16-F10 tumors, obesity did not increase the growth of MC38 tumors, and both obese and nonobese mice responded to anti–PD-1 treatment (Supplemental Figure 3A). However, infiltration of CD3^+^ T cells was decreased by obesity and restored upon anti–PD-1 treatment (Supplemental Figure 3B). Interestingly, and in line with the immunogenicity of the MC38 model, antigen-specific CD8^+^ T cell infiltration was increased by PD-1 blockade in both obese and nonobese mice (Supplemental Figure 3B). Although obesity has been shown to promote differentiation and activation of tumor-associated myeloid-derived suppressor cells (MDSC) (16), we did not observe any difference in tumor-infiltrating MDSC between obese and nonobese mice in either B16-F10 or MC38 tumors (data not shown). These results suggest that the impact of obesity on response to immune checkpoint inhibition may be context dependent.

To evaluate whether the effect of obesity on response to ICI was restricted to males, we performed PD-1 blockade experiments on female mice injected with B16-F10 tumor cells. As observed in males, tumor growth was increased in obese compared with nonobese female mice, though the difference was not significant in this experiment (*P* = 0.051) (Supplemental Figure 4A). However, whereas in males, efficacy of anti–PD-1 blockade was restricted to obese mice, both obese and nonobese females responded to anti–PD-1 treatment. In addition, anti–PD-1 treatment was more impactful in nonobese females, which displayed increased infiltration of CD3^+^ and tumor antigen-specific CD8^+^ T cells, whereas this was not observed in obese females (Supplemental Figure 4B). These results suggest that obesity has a sex-dependent effect on response to ICI.

Since the gut microbiota is known to modulate the response to immunotherapy (17) and to be modulated by obesity (18) and sex (19), we examined the composition of the gut microbiota in obese or nonobese male and female mice. We observed differences in the relative abundance in microbiota species between the different groups, but no substantial difference was observed in this model between nonobese males, which did not respond to anti–PD-1, and nonobese females, which were sensitive to anti–PD-1 treatment (Supplemental Figure 5). Moreover, although obese males, obese females, and nonobese females all responded to anti–PD-1 treatment, they displayed different compositions of microbiota species. We specifically examined the abundance of bacterial species that have been associated with a better response to immunotherapy (20–22), but we did not observe any correlation with anti–PD-1 efficacy. These results suggest that the gut microbiota is not a major contributor to the effect of obesity on the efficacy of PD-1 blockade in this model.

### Obesity modulates estrogen receptor signaling in the tumor.

**Figure 2:**
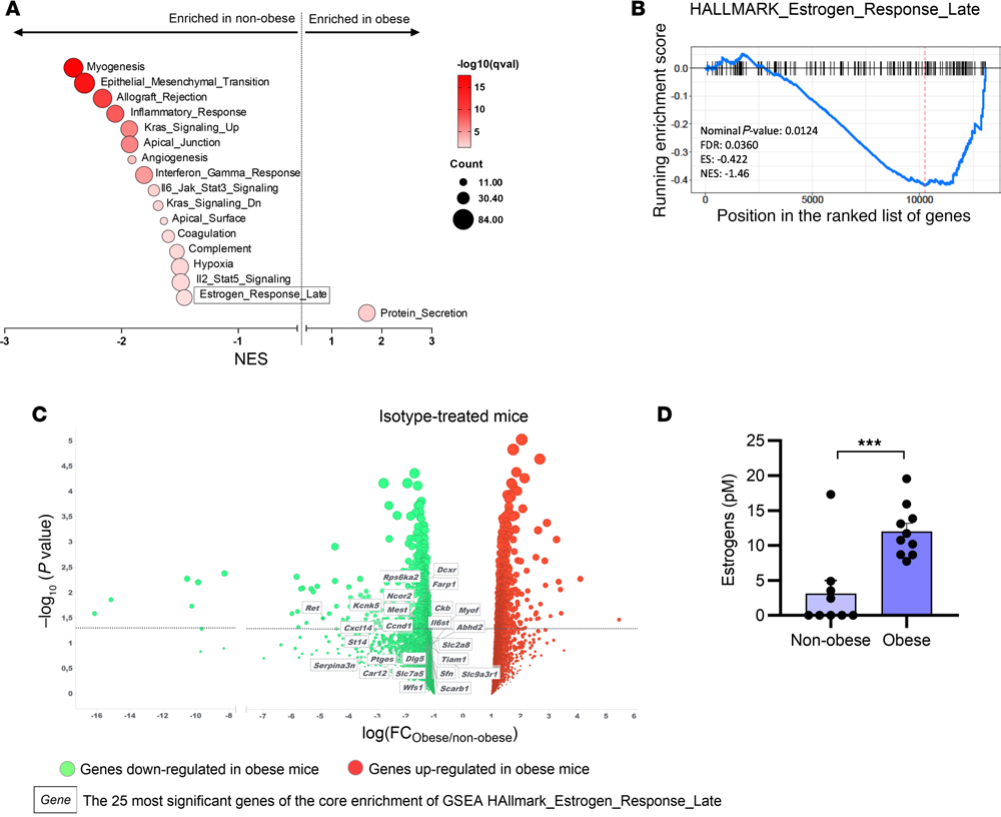
Obesity modulates estrogen signaling at the tumor site. Male C57BL/6 mice fed with a Western diet to induce obesity or with a control diet were subcutaneously injected with B16-F10 tumor cells. After the development of palpable tumors, mice received either anti–PD-1 or isotype control, and at day 16, tumors were collected and analyzed for gene expression (*n* = 3/group). (**A**) GSEA of RNA-sequencing data from B16-F10 tumors from isotype-treated mice, showing hallmarks significantly enriched in obese or nonobese males (*n* = 3/group). (**B**) GSEA plot of the Hallmark “Estrogen_Response_Late,” comparing obese males and nonobese males (*n* = 3/group). The list of genes is in the Supporting Data Values file. (**C**) Volcano plots of RNA-sequencing data from B16-F10 tumors from isotype-treated mice (*n* = 3/group). Red circles and green circles represent genes upregulated or downregulated, respectively, in obese compared with nonobese males. The 25 most significant genes from the core enrichment of the GSEA Hallmark “Estrogen_Response_Late” are labeled. (**D**) Levels of estrogens measured in the plasma of nonobese and obese males by evaluating the activity of estrogen receptor (ER) with a cell-based reporter assay (*n* = 9–10/group). Unpaired 2-tailed Student’s *t* test was used. ***P < 0.001. Data are depicted as mean ± SEM.

To investigate the mechanisms behind the increased efficacy of anti–PD-1 in obese male mice, we compared the gene expression profile of B16-F10 tumors in obese and nonobese isotype-treated mice. This analysis was done in the absence of anti–PD-1 treatment to identify any baseline effects of obesity that could explain why obese mice are more sensitive to anti–PD-1 treatment. Transcriptomic analysis revealed enrichment of gene sets related to carcinogenesis and inflammatory processes in nonobese mice compared with obese mice (Figure 2A). These observations are in line with the increased tumor growth observed in obese mice. Interestingly, we identified a signature of genes related to the response to estrogens that were differentially regulated between nonobese and obese mice (Figure 2, A and B). This suggests that several estrogen-related genes were enriched in nonobese males compared with obese males. This result was supported by differential gene expression analysis (Figure 2C) and qPCR analysis (Supplemental Figure 6), verifying the downregulation of the most significant differentially expressed genes of the estrogen response signature in obese males. Although it is known that the estrogen production is increased in adipose tissue (12), this is to our knowledge the first time that a different expression profile of estrogen-regulated genes has been observed within the tumor of obese versus nonobese males. Since estrogens act as transcription factors that can either promote or repress the expression of their target genes (23), the high levels of estrogens in obese males may repress the expression of these estrogen-related genes. Overall, these transcriptional analyses revealed that estrogen signaling within the tumor differs between obese and nonobese males. To determine whether obesity also modulates estrogen metabolism systemically, we measured plasma estrogen levels by assessing the activity of estrogen receptors in a cell-based reporter assay and found that obese males displayed higher levels of estrogens than nonobese males (Figure 2D), consistent with previous findings in humans (24–26). Since sex disparities occur in immune responses, and specific immunomodulatory properties have been attributed to sex hormones, especially estrogens (27), we hypothesized that estrogens could be an obesity-derived factor modulating the sensitivity to PD-1 blockade in male mice.

### Inhibition of 17β-estradiol synthesis reduces efficacy of anti–PD-1 treatment in obese males.

**Figure 3:**
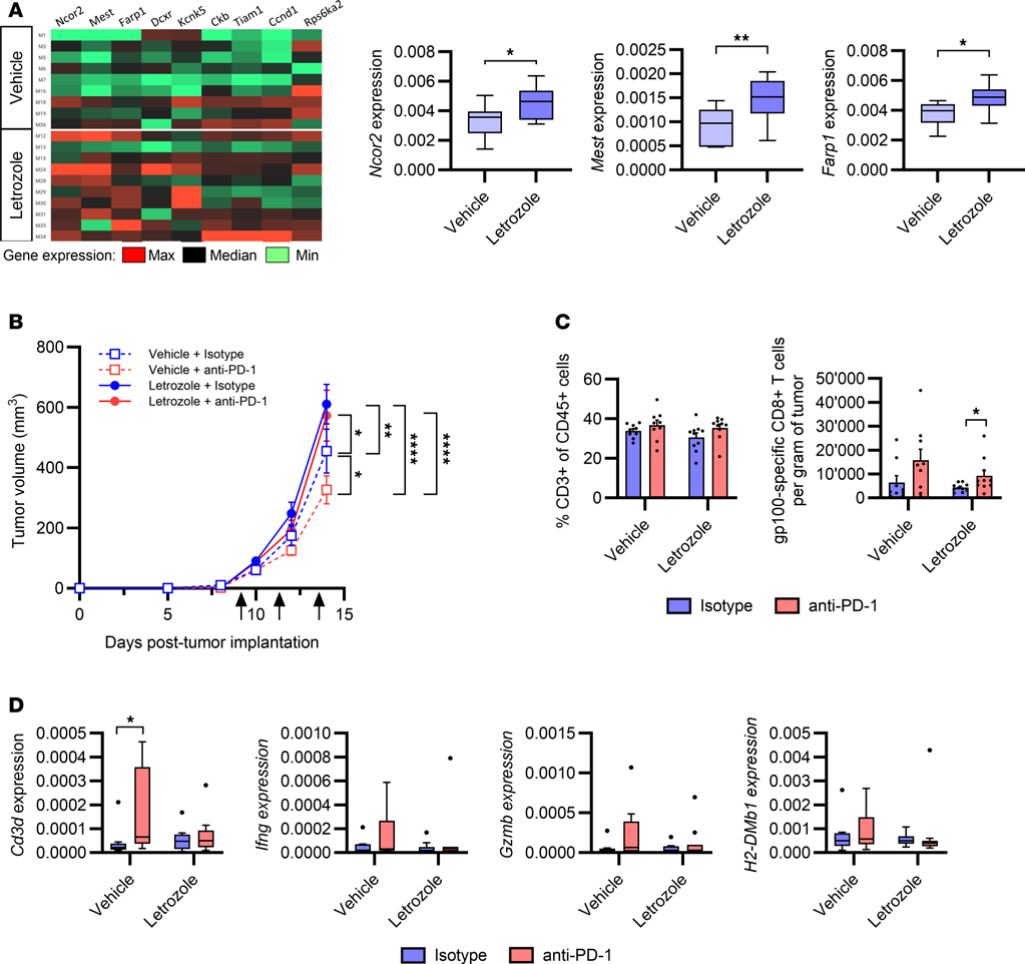
Inhibition of 17β-estradiol synthesis in obese males reduces the efficacy of anti–PD-1. Male C57BL/6 mice were fed with a Western diet to induce obesity. Daily treatment with the aromatase inhibitor letrozole was started 4 weeks before subcutaneous injection of B16-F10 tumor cells. Letrozole treatment was continued until the end of the experiment. After the development of palpable tumors, mice received either anti–PD-1 or isotype control. (**A**) Expression of the 9 most significant genes from the GSEA Hallmark “Estrogen_Response_Late,” measured by qPCR in isotype-treated mice (*n* = 9–10/group). Unpaired 2-tailed Student’s *t* test was used. *P < 0.05, **P < 0.01. Data are depicted as a heatmap and as box plots for 3 representative genes. Box plots show the interquartile range (IQR), median (line), and the most extreme values within 1.5 × IQR (whiskers). Points beyond the whiskers represent outliers. (**B**) Tumor growth of vehicle-treated (dotted line) or letrozole-treated (solid line) mice receiving anti–PD-1 (red) or isotype control (blue). Black arrows indicate anti–PD-1 or isotype treatment (*n* = 9–10/group). Two-way ANOVA with Tukey’s post hoc test was used. *P < 0.05, **P < 0.01, ****P < 0.0001. (**C**) Infiltration of CD3^+^ cells and gp100-specific CD8^+^ T cells in tumors, quantified by flow cytometry (*n* = 9–10/group). Unpaired 2-tailed Student’s *t* test was used. *P < 0.05. (**B** and **C**) Data are depicted as mean ± SEM. (**D**) Expression of selected immune genes, measured by qPCR (*n* = 9–10/group). Data are depicted as Tukey box plots and Mann-Whitney test was used. *P < 0.05.

The 17β-estradiol (E2) hormone is the main active form of estrogens and is converted from testosterone by the aromatase (CYP19A1) enzyme. Since aromatase is highly expressed in adipocytes, the adipose tissue is one of the main sources of estrogens in males (12). To investigate the impact of estrogens on the efficacy of anti–PD-1 treatment, obese males were treated with letrozole, an aromatase inhibitor, before subcutaneous injection of B16-F10 tumor cells. Transcriptomic analysis of the tumors showed a higher expression of several genes from the estrogen signature after letrozole treatment (Figure 3A). The upregulation of the estrogen target genes in letrozole-treated mice led to an expression profile that was similar to what was observed in nonobese males when compared with obese males, verifying the efficacy of letrozole treatment in affecting estrogen signaling. Letrozole- or vehicle-pretreated obese mice were then treated with anti–PD-1 or isotype control antibodies after development of palpable tumors. In vehicle-treated obese mice, we verified a decrease in tumor growth upon anti–PD-1 treatment (Figure 3B). In contrast, anti–PD-1 was not efficient in letrozole-treated mice, in which tumor growth was strongly increased compared with the vehicle-treated groups, independently of anti–PD-1 treatment. We excluded any direct proliferative effect of letrozole on B16-F10 cells (data not shown). We did not observe a significant increase in CD3^+^ T cell infiltration in response to PD-1 blockade in both groups, but anti–PD-1 treatment was associated with increased numbers of antigen-specific CD8^+^ T cells in letrozole-treated mice (Figure 3C). Gene expression analysis revealed higher expression of *Cd3d* in vehicle-treated mice in response to anti–PD-1, and a trend toward increased expression of other immune-related genes was observed (Figure 3D). In contrast, letrozole-treated mice displayed similar expression levels of immune-related genes, independently of anti–PD-1 treatment. This observation was associated with a trend toward higher numbers of antigen-specific, CD44^+^, and CD69^+^ CD8^+^ T cells in vehicle-treated mice receiving anti–PD-1 (Figure 3C and Supplemental Figure 7). Collectively, these results suggest that aromatase-derived estrogens may favor the antitumor immune response in obese males and their sensitivity to anti–PD-1 treatment. Since estrogens appeared to determine anti–PD-1 efficacy in obese mice, we investigated the impact of E2 administration in nonobese male mice. Oral administration of E2 increased the levels of circulating estrogens in nonobese mice (Supplemental Figure 8A). However, this did not affect the efficacy of anti–PD-1 treatment, since tumor growth and immune cell infiltration were similar in E2-treated and vehicle-treated groups (Supplemental Figure 8, B and C). We hypothesize that, in addition to estrogens, obesity-associated hormonal modulation affects other steroid hormones, which may also have immunomodulatory properties. This suggests that estrogens are required but not sufficient for males to respond to PD-1 blockade in this model.

### Adipocyte-derived estrogens increase the antitumor immune response by stimulating antigen-presenting cells.

**Figure 4:**
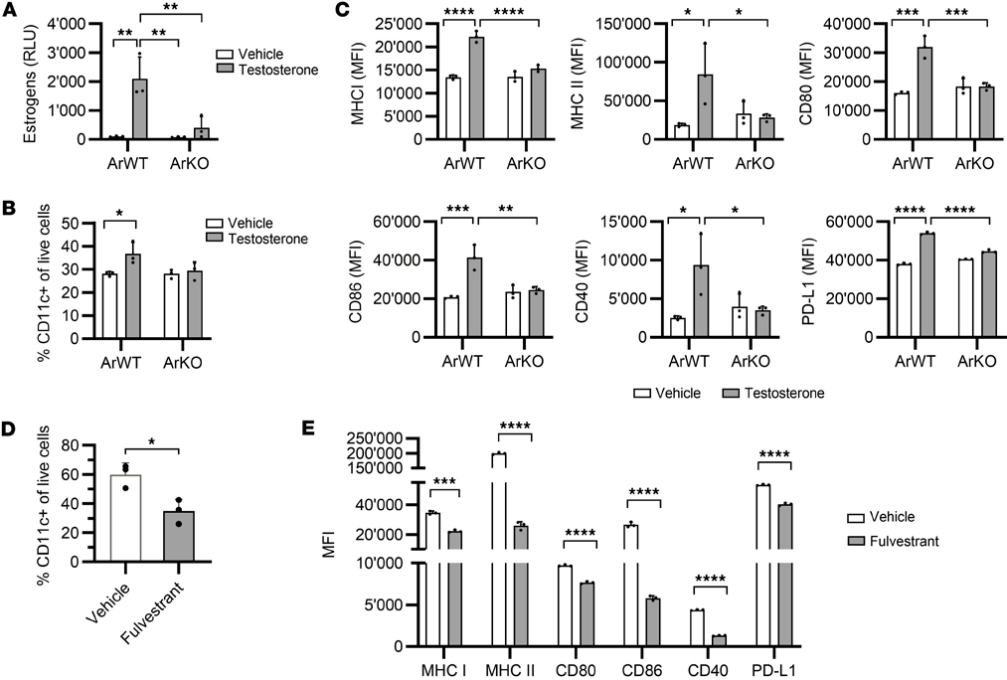
Adipocyte-derived estrogens enhance the differentiation of dendritic cells. (**A**–**C**) Adipocytes expressing the aromatase enzyme (ArWT) and aromatase-knockout adipocytes (ArKO) were generated and exposed to testosterone or vehicle (DMSO) for 24 hours. Adipocyte-conditioned supernatant was then collected. (**A**) Estrogen levels measured in adipocyte-conditioned media after incubation with testosterone (10 μM) or vehicle. Data are depicted as relative light units (RLU) and represent the activation level of ERs in a cell-based reporter assay. (**B**) Bone marrow progenitors from C57BL/6 male mice were differentiated for 6 days in a hormone-free medium supplemented with supernatant from ArWT or ArKO adipocytes, previously incubated with testosterone (0.1 μM) or vehicle. The percentage of CD11c^+^ cells was measured by flow cytometry at day 6. (**C**) Day 6 immature BMDC were stimulated with TNF-α for 24 hours, and MHC I, MHC II, CD80, CD86, CD40, and PD-L1 expression was analyzed by flow cytometry. (**A**–**C**) One-way ANOVA with Tukey’s multiple comparisons test was performed. *P < 0.05, **P < 0.01, ***P < 0.001, ****P < 0.0001. (**D** and **E**) Bone marrow progenitors from C57BL/6 male mice were differentiated during 6 days in a standard medium supplemented with fulvestrant. (**D**) The percentage of CD11c^+^ cells was measured by flow cytometry at day 6. (**E**) Day 6 immature BMDC were stimulated with TNF-α for 24 hours, and the activation profile was analyzed by flow cytometry. (**D** and **E**) Unpaired 2-tailed Student’s *t* test was used. *P < 0.05, ***P < 0.001, ****P < 0.0001. (**A**–**E**) All data are depicted as mean ± SD and are representative of 3 independent experiments.

We next investigated how estrogens produced by the adipose tissue modulated antitumor immunity using an in vitro model of adipocytes derived from human adipose stem cells. To achieve this, we generated aromatase-knockout (ArKO) adipocytes by disrupting the *CYP19A1* gene that encodes the aromatase enzyme (Supplemental Figure 9A). After incubation in the presence of testosterone, wild-type adipocytes expressing the aromatase enzyme (ArWT adipocytes) produced estrogens, whereas ArKO adipocytes did not (Figure 4A and Supplemental Figure 9B). Since adipocytes are capable of converting testosterone into other androgens through the action of different enzymes (28), we analyzed the concentration of dihydrotestosterone and androstenedione and found that both types of adipocytes produced similar levels of androgens when incubated with testosterone (Supplemental Figure 9C). This suggests that the main difference between ArWT and ArKO adipocytes in this assay is their capacity to produce estrogens.

Since we observed that genes related to antigen presentation were enriched in response to anti–PD-1 treatment in obese males (Supplemental Figure 1), we examined whether adipocyte-derived estrogens have a direct stimulatory effect on antigen-presenting cells. We generated bone marrow–derived dendritic cells (BMDC) in a hormone-free medium supplemented with supernatants from ArWT or ArKO adipocytes. The percentage of CD11c^+^ dendritic cells was increased in differentiated BMDC cultured with supernatants from testosterone-treated ArWT adipocytes but not from ArKO adipocytes (Figure 4B and Supplemental Figure 10). We then stimulated these BMDC with TNF-α and found that BMDC generated in the presence of supernatant from testosterone-treated ArWT adipocytes had higher surface levels of MHC class I and class II; CD80, CD86, and CD40 costimulatory molecules; and PD-L1 (Figure 4C). This was not the case with ArKO adipocyte supernatant, demonstrating that adipocyte-derived estrogens enhance the differentiation of bone marrow progenitors into dendritic cells and their activation.

To verify if ER signaling was the main mechanism responsible for the enhanced activation profile of BMDC in this assay, we generated BMDC in a standard medium with the addition of fulvestrant, which selectively downregulates ER. After differentiation, we found that the percentage of CD11c^+^ cells was reduced in the presence of fulvestrant, verifying the importance of ER signaling in BMDC differentiation (Figure 4D). Following stimulation with TNF-α, BMDC generated with fulvestrant showed a lower level of activation characterized by a reduced expression of MHC class I and II; CD80, CD86, and CD40 costimulatory molecules; as well as PD-L1 (Figure 4E). We also investigated the involvement of the 2 isoforms of the ER, ERα and ERβ, in the estrogen-dependent differentiation of BMDC by testing 2 selective ER antagonists, the ERα-specific antagonist methyl-piperidino-pyrazole (MPP) and the ERβ-specific antagonist 4-[2-Phenyl-5,7-bis(trifluoromethyl)pyrazolo[1,5-a]pyrimidin-3-yl]phenol (PHTPP). BMDC differentiation was not significantly affected by ER antagonists, but the activation profile of BMDC was impaired in the presence of MPP (Supplemental Figure 11). However, PHTPP treatment did not affect BMDC activation, indicating that ERα signaling, rather than ERβ, is required for the differentiation of functional BMDC.

**Figure 5:**
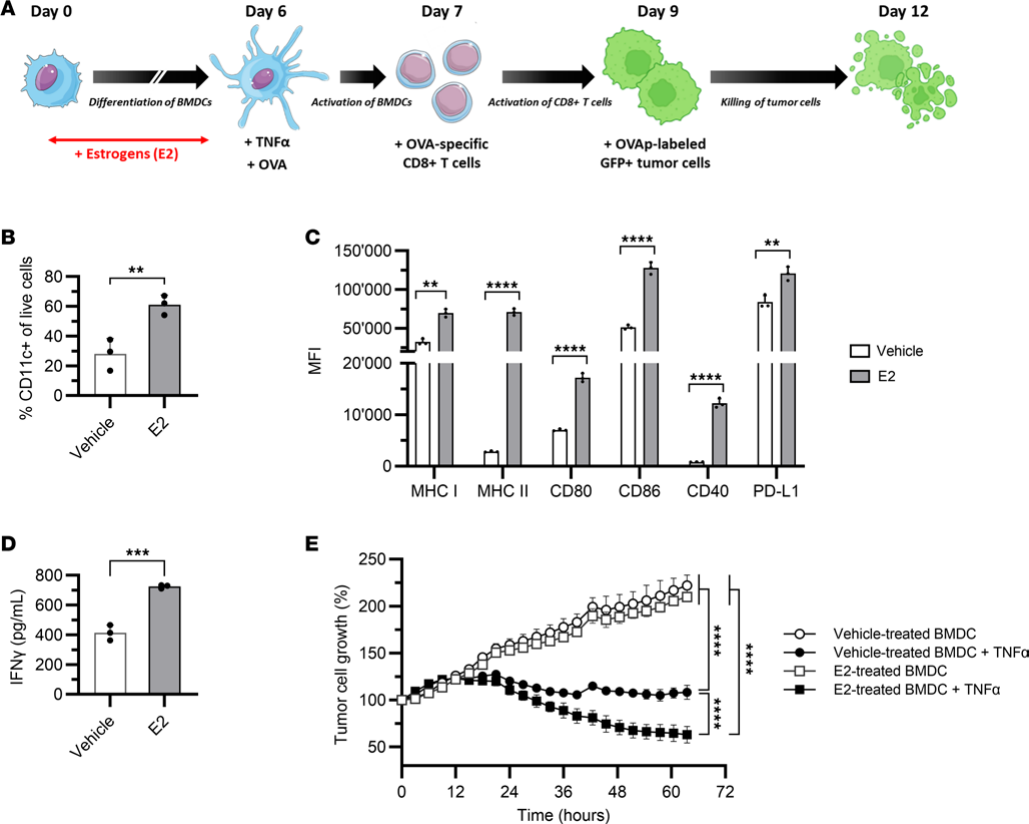
17β-Estradiol increases the antitumor immune response by stimulating antigen-presenting cells. Bone marrow progenitors from C57BL/6 male mice were differentiated for 6 days in a hormone-free medium supplemented with 17β-estradiol (E2, 1 nM). At day 6, BMDC were stimulated with TNF-α and the ovalbumin (OVA) protein. After 24 hours, OVA-specific CD8^+^ T cells were added to the BMDC. After 48 hours, GFP^+^ Renca cells were loaded with the OVA peptide and added to the coculture. (**A**) Representation of the antigen-specific cytotoxicity assay. (**B**) The percentage of CD11c^+^ cells was measured by flow cytometry at day 6. (**C**) The activation profile of stimulated BMDC was analyzed at day 7 by flow cytometry. (**D**) IFN-γ production by OVA-specific CD8^+^ T cells after 48 hours of coculture with BMDC was quantified by ELISA. (**B**–**D**) Unpaired 2-tailed Student’s *t* test was used. **P < 0.01, ***P < 0.001, ****P < 0.0001. (**E**) Growth of GFP^+^ Renca cells was monitored by live cell imaging between day 7 and day 12. Negative controls using unstimulated BMDC (no TNF-α stimulation) were used. Two-way ANOVA *P* value is shown: ****P < 0.0001. (**B**–**E**) Data represent the average of 3 independent experiments and are depicted as mean ± SD.

Next, we investigated whether the effects of estrogens on BMDC modulate the antitumor immune response. We conducted an in vitro antigen-specific cytotoxicity assay including BMDC, antigen-specific CD8^+^ T cells, and antigen-labeled tumor cells (Figure 5A). We verified that E2 had a stimulatory effect on BMDC differentiation and activation, as E2-treated BMDC exhibited a higher percentage of CD11c^+^ cells and upregulated expression of MHC and costimulatory molecules (Figure 5, B and C). After incubation with TNF-α and OVA, BMDC were cultured with OVA-specific CD8^+^ T cells isolated from OT-I mice, to assess their capacity to process and present antigens. We observed higher production of IFN-γ when CD8^+^ T cells were cultured with E2-treated BMDC, showing that BMDC generated in the presence of E2 were more efficient in priming antigen-specific CD8^+^ T cells (Figure 5D). To finally assess antigen-specific T cell functionality, we pulsed GFP^+^ tumor cells with the MHC class I–restricted OVA peptide and incubated them with BMDC and CD8^+^ T cells. Tumor cell growth was impaired when BMDC were generated in the presence of E2 (Figure 5E). We excluded any direct effect of estrogens on tumor cells, as tumor cell growth was similar in the presence or absence of E2 in the medium (Supplemental Figure 12). Together, these results demonstrate the impact of adipocyte-derived estrogens on the generation of functional dendritic cells through enhancement of their capacity to activate antigen-specific T cells and promote tumor cell killing.

### High levels of estrogens are associated with improved survival in men with melanoma treated with ICI.

**Figure 6:**
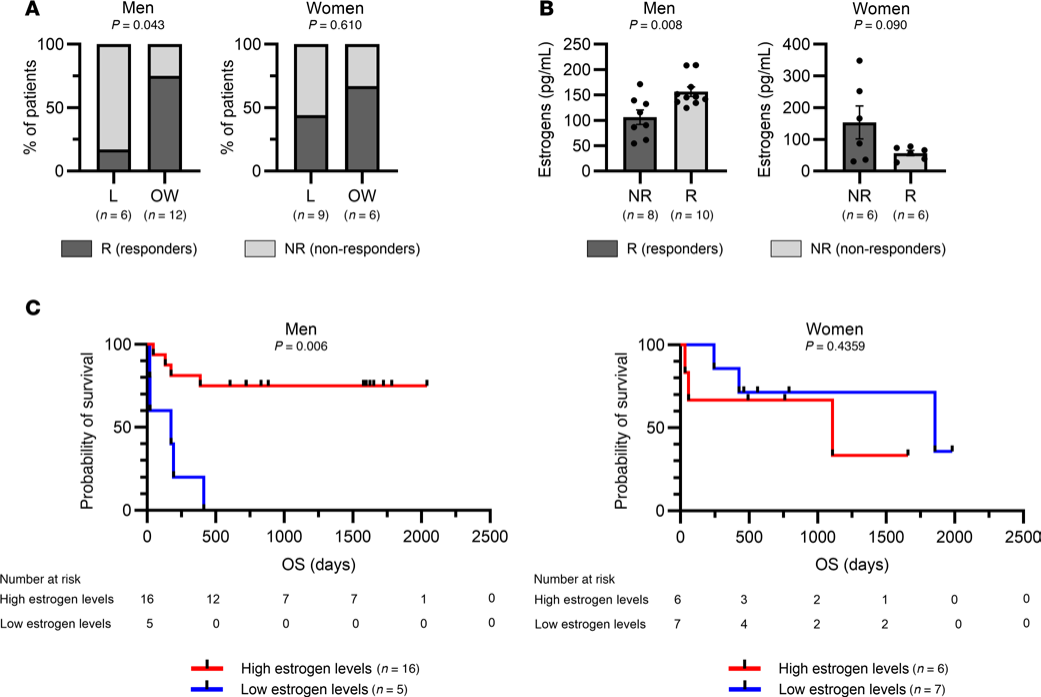
High levels of estrogens are associated with improved survival in men with melanoma treated with ICI. Clinical data and serum samples were collected from patients with melanoma treated with ICI. (**A**) Proportion of responders (R) and nonresponders (NR) among lean (L; BMI < 25 kg/m^2^) or overweight/obese (OW; BMI ≥ 25 kg/m^2^) patients, with clinical response defined by RECIST criteria (nonresponders = progressive disease, responders = complete response, partial response, and stable disease). Fisher’s exact test *P* values are shown. (**B**) Levels of estrogens measured in the serum of patients stratified by clinical response. Unpaired 2-tailed Student’s *t* test *P* values are shown. Data are depicted as mean ± SEM. (**C**) Overall survival (OS) of patients with high versus low levels of estrogens. Levels of estrogens were categorized as high or low compared with the median concentration of estrogens in L men (left) or L women (right). Mantel-Cox test *P* values are shown.

To explore the translational aspect of our findings, we studied the relationships between BMI, estrogen levels, and clinical response in a cohort of patients with melanoma treated with ICI as the first, second, or third line of treatment (Supplemental Table 1). Patients were categorized as lean (BMI < 25 kg/m^2^) or overweight/obese (BMI ≥ 25 kg/m^2^), and according to their clinical response they were classified as nonresponders (progressive disease) or responders (complete response, partial response, and stable disease) according to Response Evaluation Criteria In Solid tumors (RECIST) criteria. First, we found that about 75% of overweight/obese men were responders, compared with only 17% of responders in lean patients, whereas in women no difference was observed between lean and overweight/obese patients (Figure 6A). However, survival analysis did not show increased overall survival (OS) for overweight/obese men, even though a trend was observed (Supplemental Figure 13A).

Since we concluded that estrogens could affect the response to ICI in male mice, we next investigated the relationship between estrogen levels and clinical response. Unexpectedly, estrogen levels were not significantly increased in overweight/obese patients (Supplemental Figure 13B). However, men who responded to ICI had higher levels of estrogens compared with nonresponders (Figure 6B). Interestingly, no significant association was found in women. We then categorized patients into estrogen-high and estrogen-low patients, taking as a cutoff the median concentration measured in nonobese patients. Survival analysis showed that men with high estrogen levels had a highly significant prolonged OS compared with men with low estrogen levels (Figure 6C). In women, no association between estrogen levels and OS was observed. We observed similar results when analyzing outcomes of patients who received ICI as first-line treatment only (Supplemental Figure 13C). We also examined the relationship between estrogen levels and patient OS by considering estrogen levels as a continuous variable, and we observed that the OS in men tended to increase with estrogen levels while no association was observed in women (Supplemental Figure 13D). Together, these results indicate that high levels of estrogens are associated with enhanced response to ICI in men with melanoma.

## Discussion

The development of ICI has considerably improved the outcome of patients with cancer. However, many patients do not benefit from these treatments. Recent studies suggest that, although obese patients are more at risk of developing cancer, they may be more sensitive to ICI-based therapies (5–7, 9, 29–32) and other targeted therapies (5, 33). In this study, we found that obesity was associated with a better response to ICI in males but not in females with melanoma and identified a role for estrogens in the efficacy of ICI.

First, we showed that obese mice bearing B16-F10 tumors developed larger tumors than nonobese mice, consistent with previous observations that have identified obesity as a major risk factor for cancer (34). However, when mice were treated with ICI, we observed that obese mice responded better to PD-1 blockade than nonobese mice. We uncovered transcriptomic differences within the tumors of obese versus nonobese mice following anti–PD-1 treatment. In particular, anti–PD-1 treatment in obese mice was associated with an enrichment of genes involved in antigen processing and presentation and of genes involved in the response to IFN-γ. We also observed an increased infiltration of antigen-specific CD8^+^ T cells within the tumors of obese mice. In contrast, we did not observe differences in MDSC infiltration, though these cells are known to accumulate in obesity and can promote tumor growth (16, 35). However, the mouse models in this study were implanted tumors, which exhibit a different tumor microenvironment and immune infiltrates than spontaneous tumors. Therefore, the impact of obesity needs to be further investigated in other models. Taken together, these results suggest that obesity modulates the tumor microenvironment and creates a landscape more responsive to checkpoint inhibition.

We further showed that, in mice, the enhancement of treatment efficacy by obesity was restricted to males. In patients with melanoma, we observed a similar sex-specific relationship between obesity and response to ICI. Overweight/obese men showed a better clinical response than lean patients, whereas no difference was observed in women. This sex-specific efficacy of anti–PD-1 was unclear, since previous studies on the role of obesity in the response to immunotherapy typically used only one sex (9, 13) or rarely performed sex-based analyses (9, 36). Since obesity modulates the composition of gut microbiota, which is known to affect the response to immunotherapy, we compared the abundance of bacteria species across obese and nonobese male and female mice. However, we did not observe differences in gut microbiota composition associated with a better response to anti–PD-1. Some bacterial species that have been associated with enhanced responses to immunotherapy, such as Akkermansia (20), exhibited comparable abundance in both males and females, despite the difference in their response to anti–PD-1. Therefore, we excluded gut microbiota as a major contributor to the sex-specific effect of obesity on the efficacy of anti–PD-1. However, the small sample size and the lack of posttreatment analysis may limit the impact of gut microbiota in this study.

We propose that differences in estrogen levels may contribute to the sex-specific impact of obesity on anti–PD1 efficacy, since adipose tissue is an important source of estrogens in obese males (12). Notably, estrogens have been characterized as a tumor-promoting factor in hormone-dependent cancers, such as breast cancer, in which estrogen signaling promotes the expression of proliferative and antiapoptotic genes (23). In contrast, other studies identified tumor-suppressive functions of ER signaling in cancers that are not known to be hormone dependent, such as renal and colorectal cancers (37, 38). Here, we found that estrogen target genes were enriched in the tumors of nonobese male mice compared with obese males in the absence of treatment, suggesting that these genes are repressed by the high levels of estrogens in obese males. We further showed that inhibiting estrogen production in obese males resulted in the loss of anti–PD-1 efficacy, suggesting that beyond their known pro-tumor and tumor-suppressive properties, estrogens may have a determining role in the induction of the antitumor immune response. However, pharmacological administration of estrogen to nonobese male mice was not sufficient to enhance anti–PD-1 efficacy. This suggests that other obesity-associated factors are necessary to reproduce the hormonal changes that occur in obese mice and to improve their response to anti–PD-1 treatment. In males, the obesity-associated increased production of estrogens may be associated with lower levels of androgens, which have been shown to suppress T cell activity (39). Targeting both estrogen and androgen signaling could thus potentially be an effective strategy to optimize anti–PD-1 efficacy in males. Mechanistically, we confirmed that ERα signaling was required for the efficient differentiation of murine BMDC, in line with a previous study (40). We further evidenced the increased capacity of estrogen-treated BMDC in eliciting an antigen-specific killing of tumor cells. These findings suggest that estrogens support the induction of a tumor-specific immune response through a direct action on dendritic cells. Interestingly, it was recently shown that obesity can also affect the immune phenotype of tumor-infiltrating macrophages by enhancing PD-1 expression via inflammatory cytokines (41). However, we did not observe any effect of estrogen treatment on the differentiation and activation profile of bone marrow–derived macrophages, suggesting that dendritic cells are the primary target of estrogen activity.

In men with melanoma treated with ICI, we showed a positive association of estrogen levels with improved outcome, whereas high BMI was not correlated with patient survival. Although the small sample size of this cohort limits interpretation of the results, these data suggest that estrogens could be used as a predictive factor of response to ICI in men with melanoma. It will be important to confirm this hypothesis in a larger patient cohort. In addition, further investigation into the sex-specific impact of estrogens on tumor immunity will be important for adapting therapeutic strategies. In this study, most (14/16) female patients were postmenopausal but responded to ICI-based therapies. This suggests that estrogens are not necessary for a favorable response in postmenopausal women. In men, it is possible that the positive impact of estrogens is a combination of their direct immunostimulatory properties and an indirect effect through the reduction of androgen levels, which are known to suppress T cells (39). Overall, these observations highlight the need to perform sex-specific analyses to adapt and optimize treatment for both women and men.

Since obesity is associated with important metabolic and immune dysregulation, other mechanisms beyond estrogen signaling are likely to be involved in the survival advantage of obese patients receiving ICI-based therapies. For instance, leptin has been proposed as an obesity-derived factor favoring the efficacy of ICI (9, 10). In conclusion, this study supports an important role for estrogens in the efficacy of ICI in obese males. This work encourages further investigation of estrogen signaling in the context of anticancer immunity. Indeed, the characterization of the specific context required for a beneficial effect of estrogens may help predict the clinical response of patients with cancer receiving ICI-based therapies. This would also open the path to strategies targeting hormonal pathways to improve the efficacy of current immunotherapies.

## Methods

### Sex as a biological variable.

Our study was designed by accounting for sex as a biological variable. Thus, we examined male and female animals before focusing on male mice to further investigate sex-specific effects. Our clinical study also examined male and female patients, and we performed sex-specific analysis.

### Mice.

C57BL/6J and OT-I mice were purchased from Charles River Laboratories (France) and were housed under specific pathogen–free conditions, with 12-hour light/12-hour dark cycles and at 20°C–24°C at the animal facility of the University of Geneva. All experiments, including cell implantation, treatments, and organ sampling, were performed in the morning to limit the impact of circadian rhythm. For diet-induced obesity, 4- to 5-week-old mice were fed with a Western diet (Ssniff E15746) supplemented with 0.2% cholesterol until mice showed a 50% weight increase (15 weeks for males and 28 weeks for females) (42). A matching control diet (Ssniff E157453) was used for experiments on nonobese mice.

### Cell lines.

The B16-F10 cell line (ATCC CRL-6475) was maintained in Dulbecco’s modified Eagle medium (DMEM; Gibco 41966029) supplemented with 10% fetal bovine serum (FBS; MP Biomedicals), 100 U/mL penicillin/streptomycin (Gibco 15140122), 2 mM l-glutamine (Gibco 25030024), and 1 mM sodium pyruvate (Gibco 11360039). The MC38 cell line (Sigma-Aldrich SCC172) was maintained in DMEM/F-12 (Gibco 31330038) supplemented with 10% FBS and 100 U/mL penicillin/streptomycin. Renca H2-Kb GFP cells were previously generated in our lab as described (43) and were cultured in RPMI 1640 (Gibco 21875034) supplemented with 10% FBS, 100 U/mL penicillin/streptomycin, 2 mM l-glutamine, 1 mM sodium pyruvate, and 1× minimum essential medium nonessential amino acids (Gibco, 11140035). The T47D-KB-Luc cell line (ATCC CRL-2865) was maintained in RPMI 1640 supplemented with 10% FBS, 100 U/mL penicillin/streptomycin, and 2 mM l-glutamine. For hormone deprivation experiments, RPMI 1640 was replaced by RPMI without phenol red (Gibco 11835-063), DMEM was replaced with DMEM without phenol red (Gibco A1443001), and FBS was replaced by charcoal-stripped FBS (Gibco 12676029).

### In vivo tumor experiments.

B16-F10 cells (2 × 10^5^) or MC38 cells (1 × 10^6^) were implanted subcutaneously into the right flank of C57BL/6J mice. Tumor growth was monitored every 2 days, and tumor volumes were calculated using the formula: tumor volume = length (mm) × width (mm) × height (mm) × π/6. After the development of palpable tumors, mice were treated intraperitoneally with 10 mg/kg of anti–PD-1 monoclonal antibody (clone 29F.1A12; BioXCell BE0273) or isotype control antibody (clone 2A3; BioXCell BE0089) 3 times a week. Mice were sacrificed when tumors reached 1.5 cm in diameter. Tumors were collected, processed for flow cytometry analysis, or stored at –80°C for further analyses. Blood was collected by intracardiac puncture in heparin-treated tubes (BD 365966) and centrifuged at 2,000*g* for 10 minutes at 4°C. Plasma was collected to assess steroid hormone levels.

For pharmacological inhibition of the aromatase enzyme, obese male mice were treated by daily subcutaneous injection of 20 μg of letrozole (MilliporeSigma PHR1540) or with the vehicle (0.3% hydroxypropylcellulose, 1.3% DMSO). Treatment was initiated 4 weeks before tumor injection and was maintained until the end of the experiment. Tumor injection, anti–PD-1 or isotype antibody treatment, and organ collection were performed as described in the previous paragraph.

### Tumor digestion and cell isolation for flow cytometric analysis.

At the end of each in vivo experiment, tumors were collected postmortem, and approximately 0.2 g of tumor from each sample was digested using the tumor dissociation kit (Miltenyi Biotec 130-096-730) and the gentleMACS Dissociator (Miltenyi Biotec) following manufacturer’s recommendation for tumor dissociation of soft tumors (program 37C_m_TDK_1). Tumor suspension was then filtered through a 40 μm cell strainer (Corning 15360801) and washed with 1× phosphate-buffered saline (Gibco 10010023) before proceeding to flow cytometry staining and acquisition.

Flow cytometry, tumor gene expression analysis, generation of adipocytes and adipocyte-conditioned media, generation and activation of BMDC, BMDC-mediated T cell activation, and tumor cell killing assay are described in Supplemental Methods.

### Patients with melanoma.

Patients with melanoma were treated with ICI in the oncology division of the Geneva University Hospital between June 2017 and November 2022. In total, 37 patients with melanoma receiving anti–CTLA-4 and/or anti–PD-1 as the first, second, or third line of treatment were included in the study (Supplemental Table 1). Biological samples were collected before administration of ICI. Health-related personal data were collected to segregate patients by sex and BMI (nonobese patients [BMI < 25 kg/m^2^] versus overweight/obese patients [BMI ≥ 25 kg/m^2^]). OS was determined for the whole cohort (*n* = 37). Clinical outcome was defined based on RECIST criteria as responding (complete response, partial response, and stable disease) or nonresponding (progressive disease).

### Quantification of steroid levels.

Levels of estrogens were measured in adipocyte-conditioned media, mouse plasma, and human serum with the ER reporter cell line T47D-KB-Luc. Steroids were first extracted from the samples by acetonitrile-based protein precipitation. Briefly, acetonitrile was mixed with the samples (3:1 v/v) and incubated for 10 minutes at room temperature. After centrifugation at 20,000*g* for 10 minutes at 4°C, supernatants were collected and dried into a SpeedVac concentrator (Thermo Fisher Scientific). T47D-KB-Luc cells were starved for at least 8 hours in the assay medium, consisting of a hormone-free, antibiotic-free culture medium, before being seeded at a cell density of 3.6 × 10^4^ cells/cm^2^ in a 384-well plate. After 24 hours, dry extracts of the samples were resuspended in the assay medium and added to the cells. A dose-response assay with known concentrations of E2 was also performed to use as a standard curve. After a 24-hour incubation, cells were lysed and incubated with luciferin using the Luciferase Assay kit (OZ Biosciences LUC1000), following the manufacturer’s recommendations. Luciferase activity was measured by luminescence on CLARIOstar microplate reader (BMG Labtech), and the luminescence signal was converted in some instances into E2 concentration using a 4-parameter logistic regression of the dose response.

Levels of androgens (testosterone, dihydrotestosterone, androstenedione) in adipocyte-conditioned media were assessed by mass spectrometry. Briefly, samples were purified using solid phase extraction on an OasisPrime HLB 96-Well Plate. A Vanquish UHPLC (equipped with an ACQUITY UPLC HSS T3 Column, 100 Å, 1.8 μm, 1 mm × 100 mm column) was coupled to a Q Exactive Plus Orbitrap. Separation was achieved using gradient elution over 12 minutes using water and methanol both supplemented with 0.1% formic acid (all Sigma-Aldrich) as mobile phases. Data analysis was performed using TraceFinder 4.1 (Thermo Fisher Scientific).

### Fecal DNA metagenomics.

Feces were collected from male and female mice before tumor injection and stored at –80°C. DNA was extracted using the QIAamp Fast DNA Stool Mini Kit (QIAGEN 51604) following the manufacturer’s recommendations and sent to CosmosID for shotgun sequencing. The relative abundance of bacteria species at the genus level was assessed and is detailed in the Supporting Data Values file.

### Statistics.

Graphs were made and statistical analyses were performed using GraphPad Prism software (GraphPad Software Inc.). Data were expressed as mean ± SEM for in vivo and clinical analysis or as mean ± SD for in vitro analysis. Analysis of differences between 2 groups was performed using the unpaired 2-tailed Student’s *t* test when data follow normal distribution or using the Mann-Whitney *U* test otherwise. For analysis of 3 or more groups, 1-way ANOVA tests (for 1 factor) or 2-way ANOVA tests (for 2 factors) were performed with a Tukey’s multiple-comparison correction posttest. For the analysis of clinical data, Fisher’s exact *t* test was used to compare the proportions of responders and nonresponders. Kaplan-Meier analyses were performed with the log-rank (Mantel-Cox) test. For all analyses, a *P* value of less than 0.05 was considered statistically significant.

### Study approval.

Animal studies were performed according to the Swiss federal regulations, and procedures were approved by the veterinary authorities of the Canton of Geneva, Geneva, Switzerland (license GE/26/20).

Patients with melanoma in this study were included in a research protocol approved by the Geneva ethics committee, Geneva, Switzerland (authorization numbers CCER-2016-01237 and CCER-2020-01944), and have given written informed consent to participate in this study. Reuse of samples for the current study was approved by the Geneva ethics committee (authorization number CCER-2020-01944).

### Data availability.

RNA-Seq data sets have been deposited in the National Center for Biotechnology Information’s Gene Expression Omnibus (accession number GSE297811). Supporting Data Values are included, containing all data points shown in the graphs and the values behind reported means and medians. Additional methods are provided as Supplemental Methods.

## Author contributions

CB, AP, VD, and DM supervised the study. ED, HP, AV, MB, MA, BT, CDV, and EM conducted the experiments and collected and analyzed the data. OPS provided essential materials and methodology. ED, CB, AP, and VD wrote the manuscript. CB acts as guarantor. All authors provided a critical review of the manuscript.

## Supplementary Material

Supplemental data

Supporting data values

## Version 1 06/16/2025

In-Press Preview

## Version 2 07/22/2025

Electronic publication
