# Supplementary material for: Estrogens determine the efficacy of cancer immunotherapy in obese males with melanoma
Source: JCI Insight. 2025 Jun 12;10(14):e189758. doi: 10.1172/jci.insight.189758 (PMC12288961; doi:10.1172/jci.insight.189758)
Supplement: Supplemental data [file jciinsight-10-189758-s007.pdf]

Supplemental materials and methods

Flow cytometry

Cell suspensions from either dissociated mouse organs or cell culture experiments were first incubated with Zombie Violet^{TM} viability dye (1/1'000 in PBS; Biolegend #423114) for 15 min at room temperature, then washed with FACS buffer (PBS 0.5% bovine serum albumin (BSA; PanBiotech #P06-1391500), 2% ethylenediaminetetraacetic acid (EDTA; Promega #V4231)). Cells were then incubated for 30 min at 4°C with the anti-mouse CD16/32 (1/100 in FACS buffer; Biolegend #101320) and a mix of fluorochrome-conjugated antibodies as listed below. Except when specifically indicated, the antibodies were obtained from Biolegend and diluted at 1/200 in FACS buffer. Samples were then washed three times with FACS buffer before acquisition on the NovoCyte3000 flow cytometer (Agilent Technologies) and analysis with FlowJo™ Software (v.10.8.1). Gating strategies are detailed in Supplemental Figures 2 and 10.

The flow cytometry panels used were:

Panel 1: mouse tumor phenotyping of immune cell populations: anti-CD45.2 (#109824), anti-CD19 (#115554), anti-CD3 (#100236).

Panel 2: mouse tumor phenotyping of lymphoid cell populations: for tumor antigen-specific CD8$^+$ T cell staining, the protocol was slightly modified. After incubation with Zombie Violet^{TM} viability dye, cells were first incubated with the anti-mouse CD16/32 only, for 10 min at 4°C. After one wash with PBS, cells were incubated with a tumor antigen-specific tetramer conjugate (1/10 in FACS buffer; MBL #TB-M546-1 (H-2Db gp100) for B16-F10 tumor experiments or MBL #TB-M507-1 (H-2Kb MuLV p15E) for MC38 tumor experiments) for 1 hour at 4°C. Cells were washed once with FACS buffer and incubated with the following mix of antibodies, for 30 min at 4°C: anti-CD3 (#100236), anti-CD8 (Miltenyi #130-102-805), anti-CD44 (#103047), anti-CD69 (#104506).

Panel 3: mouse BMDC phenotyping: anti-CD11c (#117331), anti-CD80 (#104729), anti-PD-L1 (#124331), anti-MHC II (Miltenyi #130-102-168), anti-CD40 (eBioscience #12-0401-82), anti-CD11b (#550993), anti-MHC I (eBioscience #17-5958-80), anti-CD86 (#105008).

Panel 4: mouse OT-I CD8+ T cell purity: anti-CD3 (#100203), anti-CD8a (#100722), anti-CD19 (#115512), anti-CD4 (#100559), anti-CD44 (#103047), anti-CD62L (#104408).

Tumor gene expression analysis

At the end of each in vivo experiment, tumors were collected post-mortem and approximately 0.1 g of tumor from each sample was snap-frozen on dry ice for subsequent RNA analysis. Total RNA was extracted from frozen tumors using TRIzol™ Reagent (Invitrogen #15596018) and following the manufacturer's recommendations. For RNA sequencing analysis, extracted RNA was purified using the RNA Clean & Concentrator-5 kit (Zymo Research #R1014), following the manufacturer's recommendations. The concentration and integrity of cleaned RNA were determined with the Qubit® RNA High Sensitivity assay (Invitrogen #Q32852) and Bioanalyzer 2100 system (Agilent Technologies), respectively. Libraries were prepared by the iGE3 genomics platform of the University of Geneva with the TruSeq Stranded mRNA kit (Illumina) using 180 ng of total RNA per sample. Library molarity and quality were assessed with the Qubit® DNA High Sensitivity assay (Invitrogen #Q32851). Libraries were then sequenced on the HiSeq 4000 System (Illumina) using a 100-bp single-end reads protocol. Quality control was performed with FastQC (v.0.11.5). Reads were mapped with the STAR aligner (v.2.7.0f) to the mouse reference genome (UCSC mm10). Biological quality control was done with PicardTools (v.2.21.6). Raw counts were obtained using HTSeq (v.0.9.1). Normalization and differential expression analysis were performed with the R package edgeR (v.3.28.1) using the Trimmed Mean of M-values (TMM) normalization method. Statistical significance was assessed with a negative binomial generalized linear model and using a quasi-likelihood F-test. Genes with a fold change > 2 and p-value < 0.05 were considered differentially expressed. Over-representation analysis (ORA) and gene set enrichment analysis (GSEA) were also performed with the edgeR package. Volcano plots were visualized with the Spotfire Analyst software (v.12.2.0; TIBCO).

For quantitative real-time polymerase chain reaction (qRT-PCR), RNA concentration was determined with a Nanodrop™ (Thermo Fisher), and reverse-transcription into cDNA was performed on 1 µg of total RNA using the Applied Biosystems™ High-Capacity cDNA Reverse Transcription Kit (Thermo Fisher #4368814). qRT-PCR was performed on QuantStudio™ 5 Real-Time PCR System

using the Applied Biosytems™ PowerUp™ SYBR™ Green Master Mix (Thermo Fisher #A25742) and the primers listed below (Eurogentec). β-actin was used as a housekeeping gene and relative gene expressions were calculated by $2^{-\Delta\Delta Ct}$ formulations.

Target	Primer	Sequence
Mouse β-actin	Forward	CACTGTCGAGTCGCGTCC
	Reverse	TCATCCATGGCGAACTGGTG
Mouse *Ncor2*	Forward	CAGCCAGCATAGAGGGACTC
	Reverse	GTAAGTAGTCCTCCTGCGCC
Mouse *Mest*	Forward	GAGTGGTGGGTCCAAGTAGG
	Reverse	ACCACACCGACAGAATCTTGG
Mouse *Farp1*	Forward	CTTCCGGTTTAGTGGTCGGA
	Reverse	TGGTGGAGTGGATCTTGCTG
Mouse *Dcxr*	Forward	TCCAGGTGTCTCAGATTGTGG
	Reverse	TATGGTTGGTCAGTGCACGTT
Mouse *Kcnk5*	Forward	GGAGAGGAGTGAGCCTGAGGAA
	Reverse	ACCATGAACACAAAGGGCGG
Mouse *Ckb*	Forward	CGTAGACAATCCGGGCCACC
	Reverse	GCTCATCACTGGGCTGGTAG
Mouse *Tiam1*	Forward	GATCCTTCCTGTACTACGCGG
	Reverse	CTGTGTCCGTCTTGGCTTTC
Mouse *Ccnd1*	Forward	TGCAGAAGGAGATTGTGCCAT
	Reverse	CTTCTGCTCCTCACAGACCTCC
Mouse *Rps6ka2*	Forward	GTAGCTCCCGAGGTCTTGAA
	Reverse	GTACTTCCCACTGCCGATCC
Mouse *Cd3d*	Forward	GTCCATTACCGAATGTGCCAG
	Reverse	AGGAGCAGAGTTGCGATGAG
Mouse *Ifng*	Forward	CGGCACAGTCATTGAAAGCC
	Reverse	TGCATCCTTTTTCGCCTTGC
Mouse *GzmB*	Forward	GCTGCTCACTGTGAAGGAAGTATAA
	Reverse	AGGGATGACTTGCTGGGTCT
Mouse *H2-DMb1*	Forward	ACCTACCCGGAAGGACAGCA
	Reverse	CCCACCTGGGACAGACTTCATT

In vivo administration of estrogens to non-obese males

Male C57BL/6 mice were treated with 2 µg/mL of 17β-estradiol (E2) or vehicle (ethanol) in drinking water for 4 weeks before subcutaneous injection into the right flank of B16-F10 tumor cells (2×10^5). E2 treatment was continued until the end of the experiment. After the development of palpable tumors, mice were treated intraperitoneally with 10 mg/kg of anti-PD-1 monoclonal antibody (clone 29F.1A12; BioXCell #BE0273) or isotype control antibody (clone 2A3; BioXCell #BE0089) three times

a week. Mice were sacrificed when tumors reached 1.5 cm in diameter. Tumors were collected, processed for flow cytometry analysis, or stored at -80°C for further analyses. Blood was collected by intracardiac puncture in heparin-treated tubes (BD #365966) and centrifuged at 2000 g for 10 min at 4°C. Plasma was collected to assess steroid hormone levels.

In vitro generation of adipocytes and adipocyte-conditioned media

Human adipose-derived cells (ASC) were kindly provided by Dr. Olivier Preynat-Seauve from the University of Geneva (Geneva, Switzerland). Cells were cultured in DMEM supplemented with 100 U/mL penicillin/streptomycin, 10 % of human platelet lysate (StemCell #06960), and 2 U/mL heparin (Sigma #3149). Knockout of the *CYP19A1* gene encoding the aromatase enzyme was performed using CRISPR/Cas9 technology on the Neon™ Transfection System (Invitrogen) and following the manufacturer's recommendations. Briefly, the RNA oligo duplex was prepared with the Alt-R Cas9 tracrRNA (IDT #1075928) and the RNA guides in the table below (or with the synthetic non-targeting control guide RNA (IDT #1072544) for negative control), and heated for 5 min at 95°C.

Design ID	Gene Symbol	Sequence
Hs.Cas9.CYP19A1.1.AA	CYP19A1	TGACCATACGAACAAGGCCG
Hs.Cas9.CYP19A1.1.AB	CYP19A1	GACCAATGAATCGGGCTATG

RNA oligo duplex was then mixed with the TrueCut™ Cas9 Protein v2 enzyme (Invitrogen #A36499) and incubated for 5-10 min at room temperature to form the ribonucleoprotein (RNP) complex. ASCs were prepared at 1.10^7 cells/mL in an antibiotic-free medium and 10 µL of cell suspension were mixed with the RNP complex and Alt-R Cas9 enhancer (IDT #1075916). Transfection was then performed on the Neon™ Transfection System using the following parameters: pulse voltage = 1400 V, pulse width = 10 ms, pulse number = 3. After a few days of culture, DNA was extracted using the QuickExtract™ DNA Extraction kit (Epicentre, #QE0905T) and PCR was performed with the primers below and the GoTaq® G2 Flexi DNA Polymerase kit (Promega #M7801) to assess the efficacy of the *CYP19A1* knockout.

Design ID	Sequence	Product length (bp)
HumanCYP19A1-F	GTTATCCCTCCTAGCTCCTTGT	409
HumanCYP19A1-R	AGGAGACCACAGAAAGCATACG	

To generate adipocytes, ASCs were plated in 12-well plates at 5.10^3 cells/cm^2. When cells reached 80-90% confluence, the culture medium was replaced by the differentiation medium that consists of the culture medium supplemented with 66 µM biotin (Sigma #B4639), 34 µM D-pantothenate (Sigma #P5155), 5 µM rosiglitazone (Sigma #557366), 1 µM dexamethasone (Sigma #D4902), 200 nM insulin (Sigma #I0516) and 250 µM 3-Isobutyl-1-methylxanthine (IBMX; Sigma #I5879). After 7 days of differentiation, the medium was switched to a maintenance medium consisting of the differentiation medium without IBMX and rosiglitazone. Adipocytes were ready to use after 5-10 days of maintenance. Differentiation was verified by staining the lipid droplets with BODIPY™ reagent (Thermo Fisher #D3922). Mature adipocytes were cultured for 24 hours in a hormone-free medium supplemented with several concentrations of testosterone (Sigma #46923) or vehicle (DMSO). Adipocyte-conditioned media were then collected, filtered, and stored at -20°C for further analysis.

In vitro generation and activation of bone marrow-derived dendritic cells

Bone marrow-derived dendritic cells (BMDC) were prepared from C57BL/6J male mice. Similar experiments were performed using female mice but a high variability across experiments was observed. We hypothesized that the hormonal fluctuations related to the menstrual cycle in females may impact their response to estrogens, thus we did not include these experiments in the manuscript. Briefly, bone marrow cells were extracted from the hind leg bones and incubated with 1X Pharm Lyse™ solution (BD #555899) for 2 min to lyse erythrocytes. Bone marrow cells were washed with PBS and seeded at 6.5×10^5 cells/cm^2 in 6-well plates in RPMI containing 10% FBS, 100 U/mL penicillin/streptomycin, 2 mM L-glutamine, 0.5 mM sodium pyruvate, 50 µM 2-mercaptoethanol (Gibco, #31350010). The medium was freshly supplemented with 20 ng/mL of GM-CSF (Peprotech #315-03) and renewed on day 2 and day 3 to induce differentiation into the dendritic cell lineage.

For experiments with adipocyte-conditioned media, BMDC differentiation was performed in a hormone-free medium consisting of the standard medium with RPMI replaced by RPMI without phenol red and FBS by charcoal-stripped FBS. The medium was freshly supplemented with 20 ng/mL of GM-CSF and 5% of adipocyte-conditioned medium previously collected and renewed on day 2 and day 3.

For experiments on estrogen receptor (ER) blockade, the standard medium was supplemented with 0.1 μM of the selective estrogen receptor downregulator fulvestrant (Selleck Chemicals #S1191), the ERα-specific antagonist methyl-piperidino-pyrazole (MPP; Tocris #1991), or the ERβ-specific antagonist 4-[2-Phenyl-5,7-bis(trifluoromethyl)pyrazolo[1,5-a]pyrimidin-3-yl]phenol (PHTPP; MedChem #HY-103456).

For hormone-deprivation experiments, BMDC differentiation was performed in a hormone-free medium consisting of the standard medium with RPMI replaced by RPMI without phenol red and FBS by charcoal-stripped FBS. The medium was freshly supplemented with 20 ng/mL of GM-CSF and 1 nM 17β-estradiol (E2; Sigma #E2257) or vehicle (ethanol) and renewed on day 2 and day 3.

On day 6, immature BMDCs were seeded in 96-well flat-bottom plates at a density of $1x10^5$ cells/well and stimulated with 40-100 ng/mL TNFα (Biolegend #575206) for 24 hours. Cell phenotype after differentiation (day 6) and after stimulation (day 7) was assessed by flow cytometry (panel 3, mouse BMDC phenotyping).

In vitro BMDC-mediated T cell activation and tumor cell killing assay

BMDC were generated in a hormone-free medium supplemented with E2 or vehicle (ethanol) as described in the previous section for hormone-deprivation experiments. On day 6, immature BMDC were seeded in 96-well flat-bottom plates at a density of $2\text{-}4x10^4$ cells/well, stimulated with 40-100 ng/mL TNFα (Biolegend #575206), and incubated with the ovalbumin protein (OVA, 25 μg/mL; Invivogen #vac-pova) for 24 hours. On day 7, spleens were collected from OT-I male mice and smashed on a 40 μm cell strainer. After 2 min of erythrocyte lysis with 1X Pharm Lyse™ solution, splenocytes were washed with PBS and OT-I $CD8^+$ T lymphocytes were isolated using the $CD8a^+$ T Cell Isolation Kit (Miltenyi #130-104-075) with the LS Columns (Miltenyi #130-042-401) and the MidiMACS™ Separator (Miltenyi #130-042-501), following the manufacturer's recommendations. Purity was checked by flow cytometry (panel 4, mouse OT-I $CD8^+$ T cell purity). After isolation, $6x10^4$ OT-I $CD8^+$ T cells were added to each well containing the BMDC. BMDC cultured without the OVA protein or

TNFα were included as negative controls. Co-culture was performed in the hormone-free medium supplemented with E2 or vehicle used for the differentiation of BMDC, in the absence of GM-CSF.

After 48 hours of co-culture, a fraction of cell culture supernatant was harvested to measure IFNγ production by ELISA (Biolegend #430801) and replaced with 7-10x10^3 Renca H2-Kb GFP cells per well. These tumor cells were previously incubated with the MHC I-restricted OVA-derived SIINFEKL peptide (2 µg/mL; Invivogen #vac-sin) for 1 hour at 37°C to evaluate antigen-specific killing of tumor cells by OT-I $CD8^+$ T cells. Tumor cell growth was monitored over several days by live cell imaging on the Incucyte® Live-Cell Analysis System (Sartorius). Servier Medical Art, licensed under CC BY 4.0, was utilized to make the schematic representation of the killing assay.

Supplemental figures

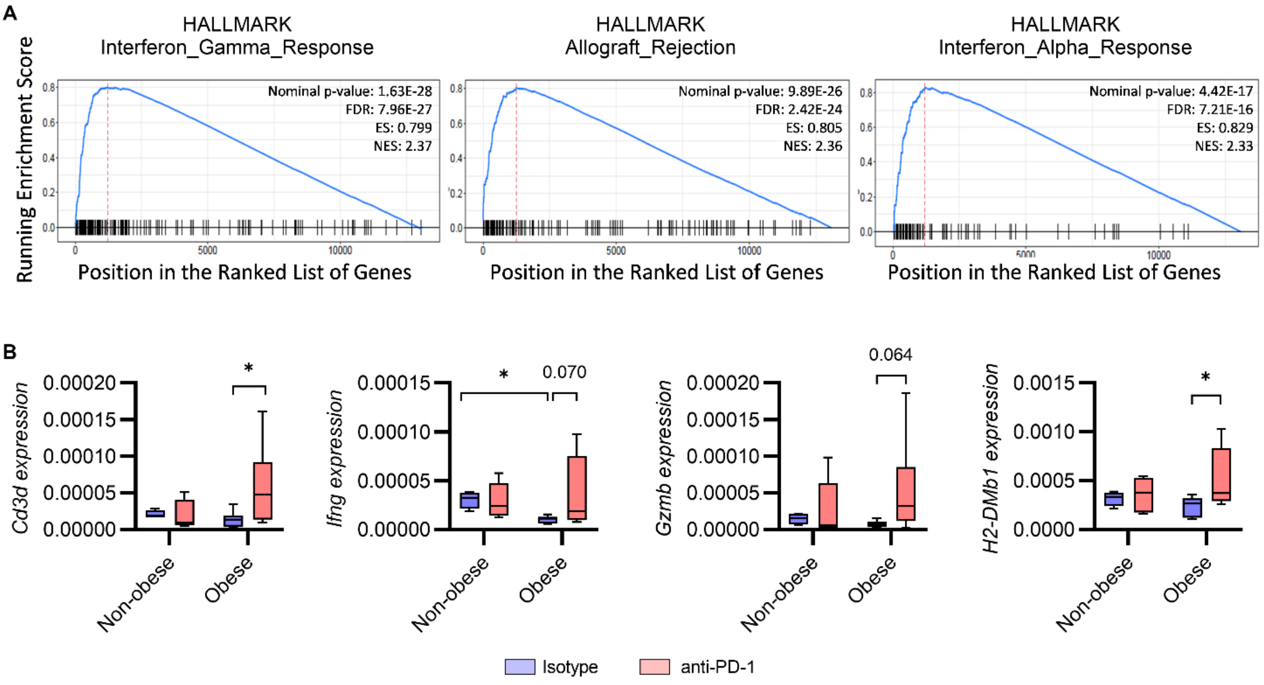

Supplemental Figure 1: Enrichment in immune-related genes in B16-F10-bearing obese male mice treated with anti-PD-1. Male C57BL/6 mice fed with a Western diet to induce obesity or with a control diet were subcutaneously injected with B16-F10 tumor cells. After the development of palpable tumors, mice received either anti-PD-1 or isotype control, and at day 16 tumors were collected and analyzed for gene expression. (**A**) GSEA plot of the 3 gene sets most significantly enriched in obese males receiving anti-PD-1 compared to isotype-treated mice (n=3/group). (**B**) Expression of immune genes, measured by qPCR (n=4-7/group). Data are depicted as Tukey boxplots and Unpaired two-tailed Student's t-test p-values are shown. *$p<0.05$.

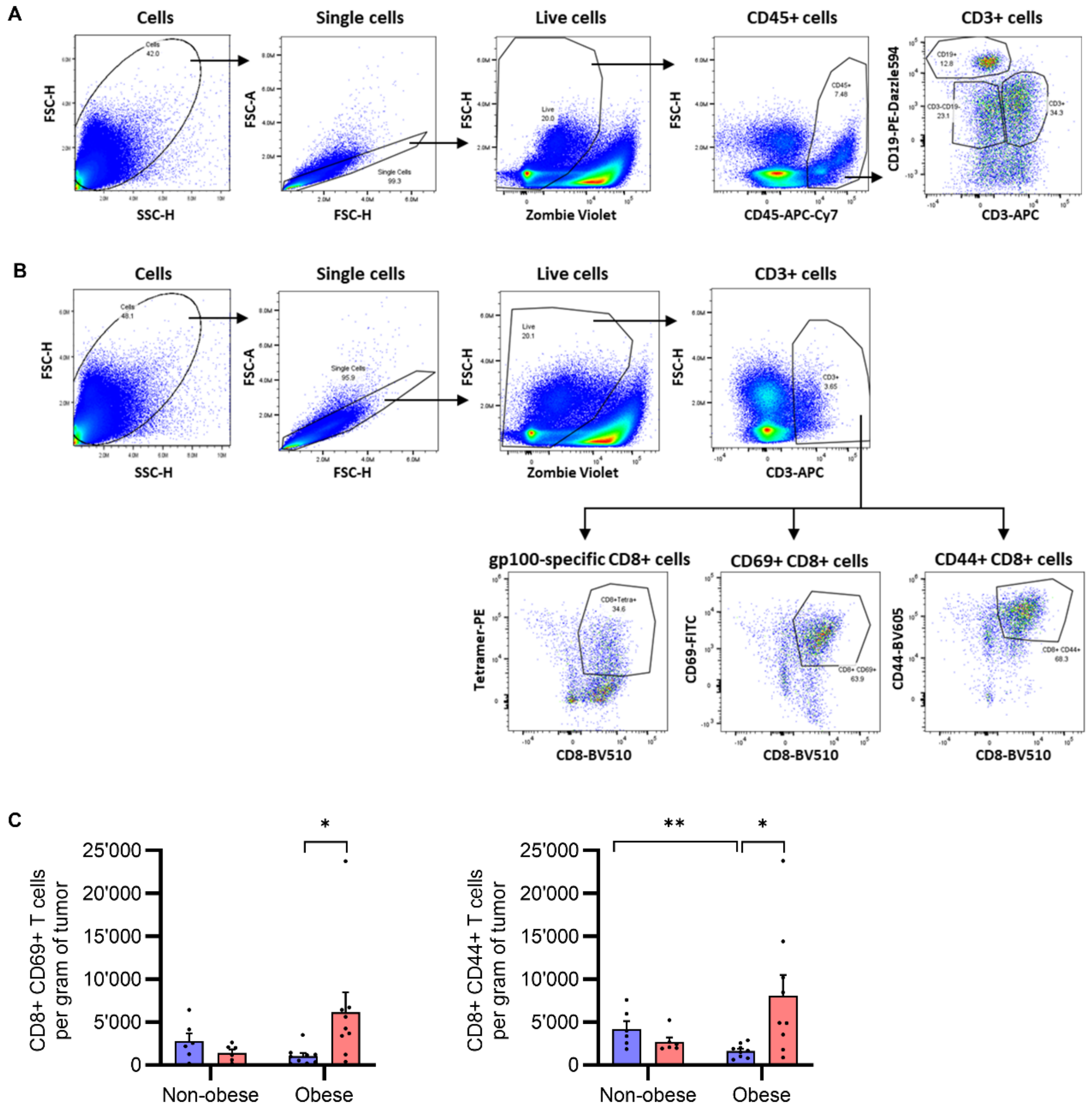

Supplemental Figure 2: Phenotyping of B16-F10-infiltrating CD8$^+$ T cells in non-obese and obese males. (**A-B**) Flow cytometry gating strategy to identify (**A**) CD3$^+$ cells and (**B**) gp100-specific, CD69$^+$ and CD44$^+$ CD8$^+$ T cells. (**C**) Number of CD8$^+$ CD69$^+$ T cells (left) and CD8$^+$ CD44$^+$ T cells (right) per gram of tumor (n=6-10/group). Unpaired two-tailed Student's t-test was used. *p<0.05, **p<0.01. Data are depicted as mean ± SEM.

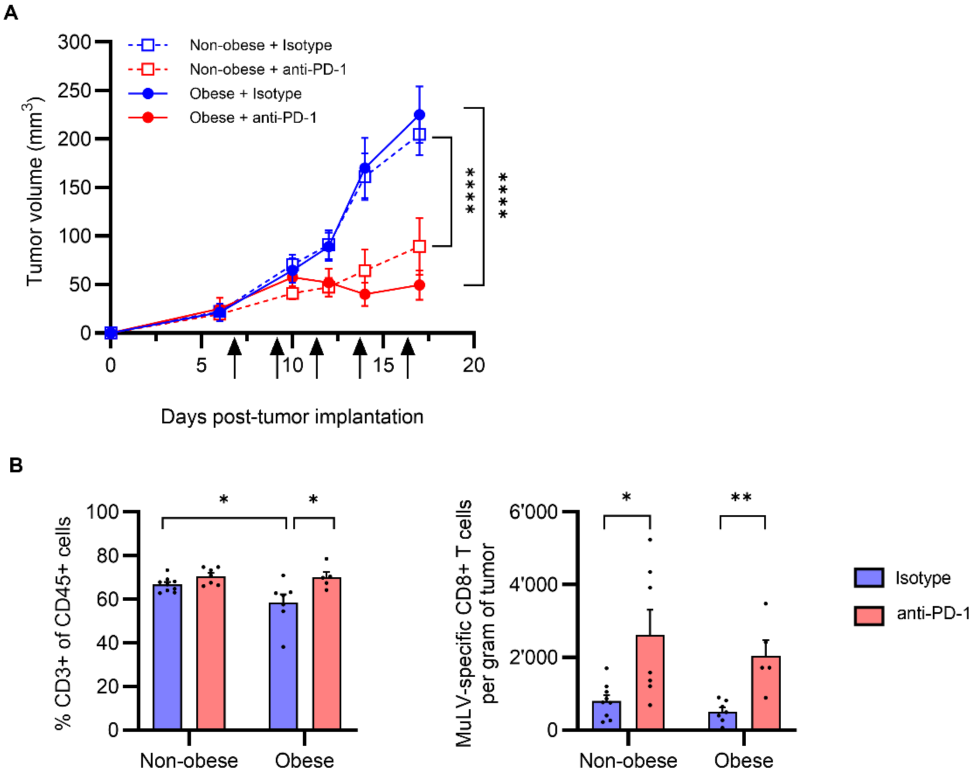

Supplemental Figure 3: Efficacy of anti-PD-1 treatment in MC38 tumor-bearing obese and non-obese male mice. Male C57BL/6 mice fed with a Western diet to induce obesity or with a control diet were subcutaneously injected with MC38 tumor cells. After the development of palpable tumors, mice received either anti-PD-1 or isotype control. (**A**) Tumor growth in non-obese (dotted line) or obese mice (solid line), receiving anti-PD-1 (red) or isotype control (blue). Black arrows indicate anti-PD-1 or isotype treatment (n=7-10/group). Two-way ANOVA with Tukey post-hoc test was used. ****$p<0.0001$. (**B**) Tumor infiltration of CD3$^+$ cells (left) and MuLV-specific CD8$^+$ T cells (right), measured by flow cytometry (n=5-9/group). Unpaired two-tailed Student's t-test was used. *$p<0.05$, **$p<0.01$, ***$p<0.001$. (**A-B**) Data are all depicted as mean ± SEM.

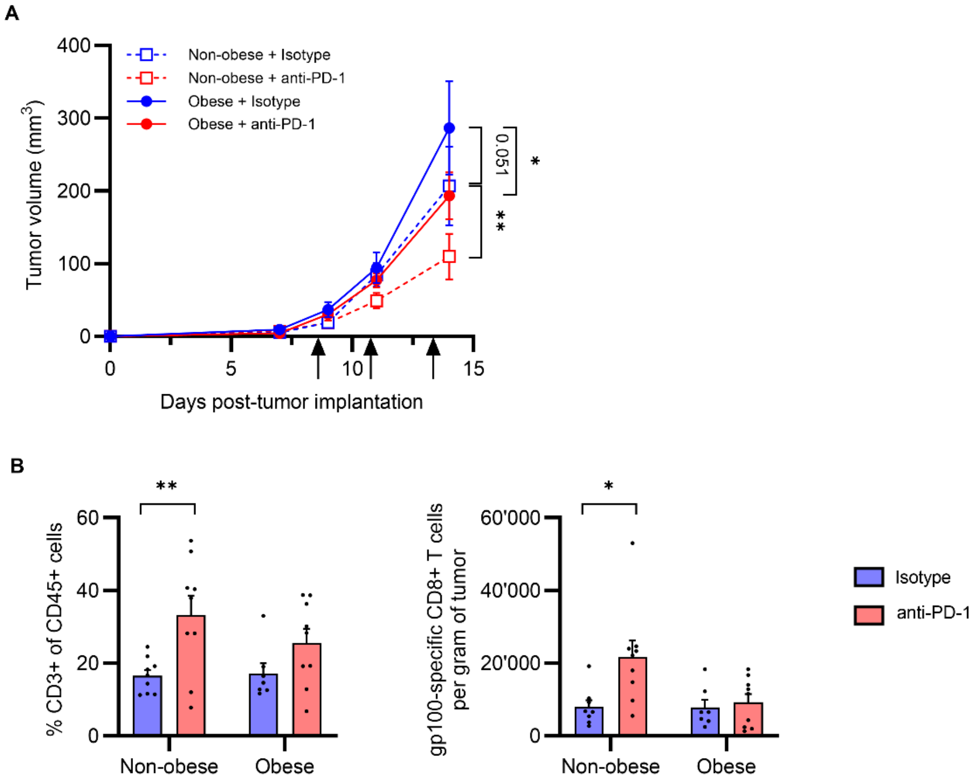

Supplemental Figure 4: Efficacy of anti-PD-1 treatment in obese and non-obese females. Female C57BL/6 mice fed with a Western diet to induce obesity or with a control diet were subcutaneously injected with B16-F10 tumor cells. After the development of palpable tumors, mice received either anti-PD-1 or isotype control. (**A**) Tumor growth in non-obese (dotted line) or obese mice (solid line), receiving anti-PD-1 (red) or isotype control (blue). Black arrows indicate anti-PD-1 or isotype treatment (n=9-10/group). Two-way ANOVA with Tukey post-hoc test was used. *p<0.05, **p<0.01. (**B**) Tumor infiltration of $CD3^+$ cells (left) and gp100-specific $CD8^+$ T cells (right), measured by flow cytometry (n=7-9/group). Unpaired two-tailed Student's t-test was used. *p<0.05, **p<0.01. Data are all depicted as mean ± SEM.

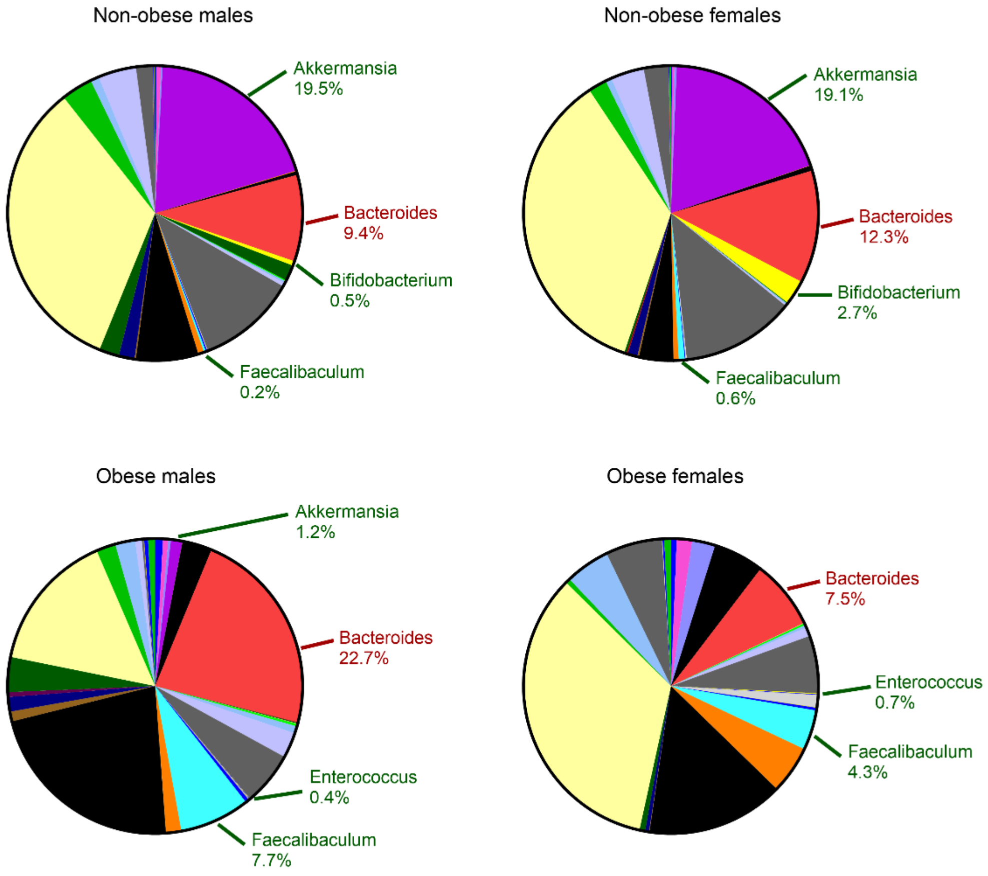

Supplemental Figure 5: Impact of obesity and sex on microbiota composition. Male and female C57BL/6 mice were fed with a Western diet to induce obesity or with a control diet for 4 months (males) or 7 months (females). Feces were collected before tumor injection. DNA was extracted from feces, sequenced, and analyzed by shotgun metagenomics to determine microbiota composition (n=3/group). The relative abundance of bacterial taxa at the genus level were plotted and the main bacteria species described as modulating the response to immunotherapy are shown (green: correlation with better response; red: correlation with lower response). The relative abundance of all bacteria species is detailed in the Supporting Data Values file.

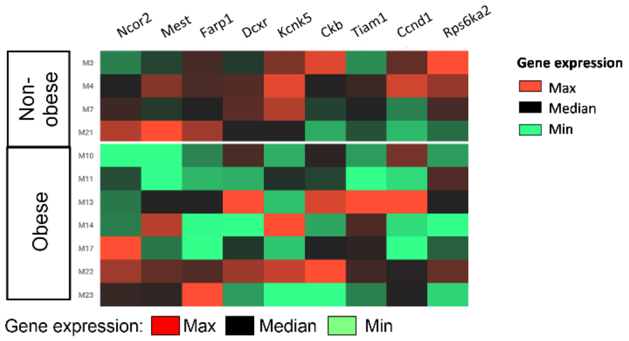

Supplemental Figure 6: Differential expression of estrogen-related genes between non-obese and obese males. Male C57BL/6 mice fed with a Western diet to induce obesity or with a control diet were subcutaneously injected with B16-F10 tumor cells. After the development of palpable tumors, mice received either anti-PD-1 or isotype control. At the end of the experiment, tumors were collected, processed, and analyzed by qPCR. The expression of the most significant genes from the Hallmark "Estrogen_Response_Late" was measured in isotype-treated mice (n=4-7/group).

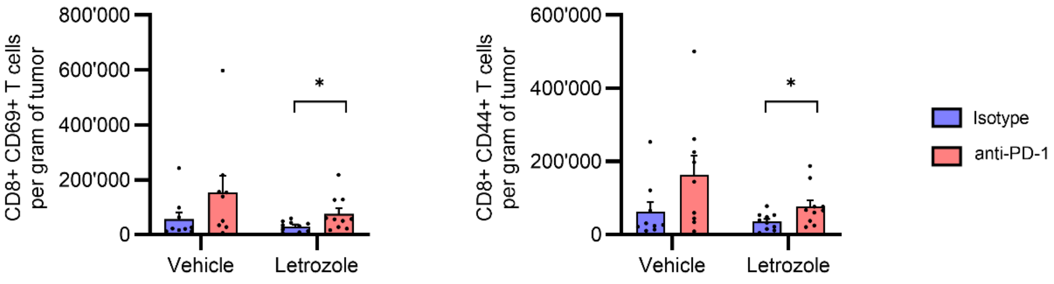

Supplemental Figure 7: Effect of letrozole on the infiltration of immune cells in B16-F10 tumors in obese males. Male C57BL/6 mice were fed with a Western diet to induce obesity. Daily treatment with the aromatase inhibitor letrozole was started 4 weeks before subcutaneous injection of B16-F10 tumor cells. Letrozole treatment was continued until the end of the experiment. After the development of palpable tumors, mice received either anti-PD-1 or isotype control. Tumor infiltration of $CD8^+$ $CD69^+$ T cells (left) and $CD8^+$ $CD44^+$ T cells (right) was measured by flow cytometry (n=9-10/group). Unpaired two-tailed Student's t-test was used. *$p<0.05$. Data are depicted as mean ± SEM.

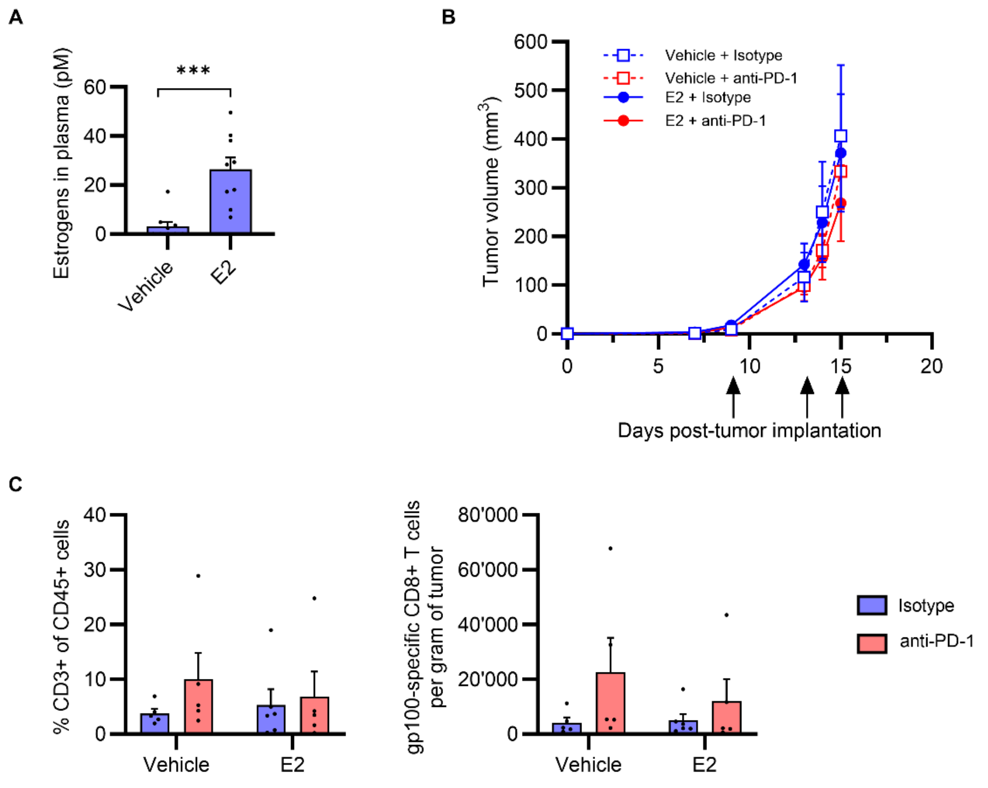

Supplemental Figure 8: Effect of E2 administration on the efficacy of anti-PD-1 treatment in non-obese males. Male C57BL/6 mice were treated with 2 µg/mL of 17β-estradiol (E2) or vehicle (ethanol) in drinking water for 4 weeks before subcutaneous injection of B16-F10 tumor cells. E2 treatment was continued until the end of the experiment. After the development of palpable tumors, mice received either anti-PD-1 or isotype control. (**A**) Levels of estrogens measured in plasma at the end of the experiment (n=9/group). Unpaired t-test was used. ***p<0.001. (**B**) Tumor growth in vehicle-treated (dotted line) or E2-treated (solid line) mice receiving anti-PD-1 (red) or isotype control (blue). Black arrows indicate anti-PD-1 or isotype administration (n=6-7/group). (**C**) Tumor infiltration of $CD3^+$ cells (left) and gp100-specific tumor antigen-specific $CD8^+$ T cells (right), measured by flow cytometry (n=5-6/group). Data are all depicted as mean ± SEM.

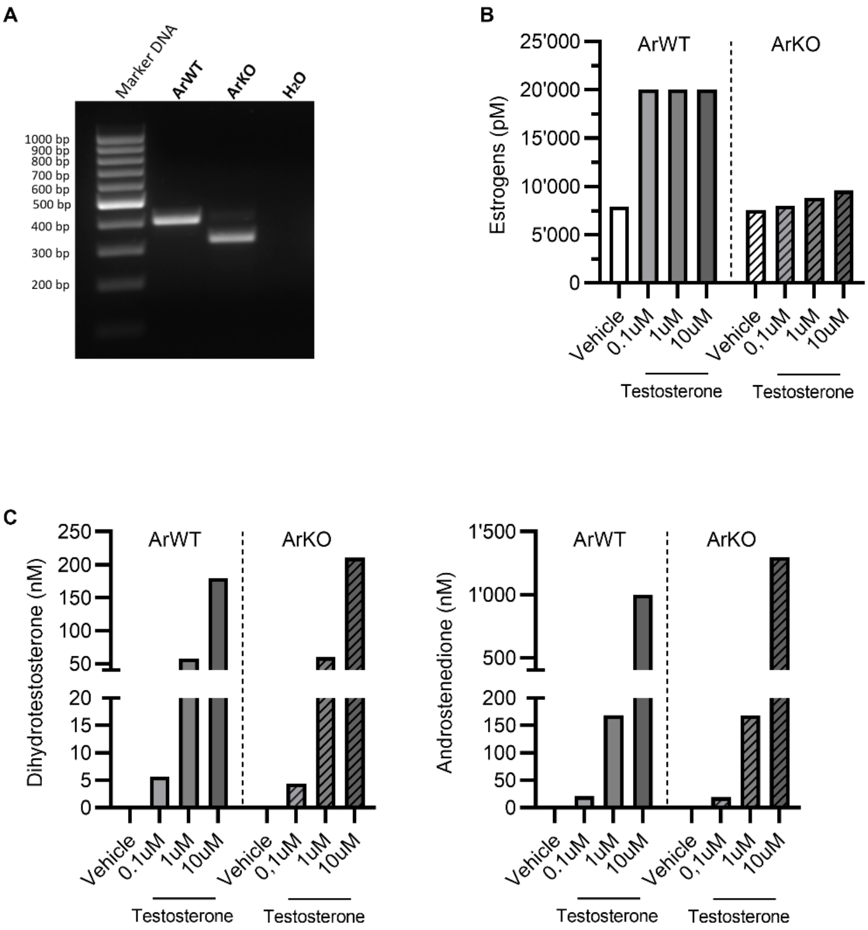

Supplemental Figure 9: In vitro generation of adipocytes converting testosterone into estrogens, depending on the expression of the aromatase enzyme. Human adipose-derived stem cells (ASC) were electroporated with a guide RNA targeting the *CYP19A1* gene and CRISPR/Cas9 technology was used to knock out the aromatase enzyme (ArKO) and were then differentiated into adipocytes. Adipocytes transfected with a synthetic non-targeting control guide RNA were used as control (ArWT). (**A**) Expression of the *CYP19A1* gene assessed by PCR on ArWT and ArKO ASC to validate knock-out of the aromatase enzyme. (**B-C**) ArWT and ArKO adipocytes were generated and incubated with testosterone (0.1-10 µM) or vehicle (DMSO) for 24 hours. Adipocyte-conditioned supernatants were collected. The levels of (**B**) estrogens, and (**C**) dihydrotestosterone and androstenedione were measured in the adipocyte supernatants. The concentration of estrogens in the supernatants from ArWT adipocytes stimulated with testosterone was outside the range of the assay (estrogen concentration higher than 20'000 pM). One experiment representative of three independent experiments for the stimulation with 10 µM of testosterone.

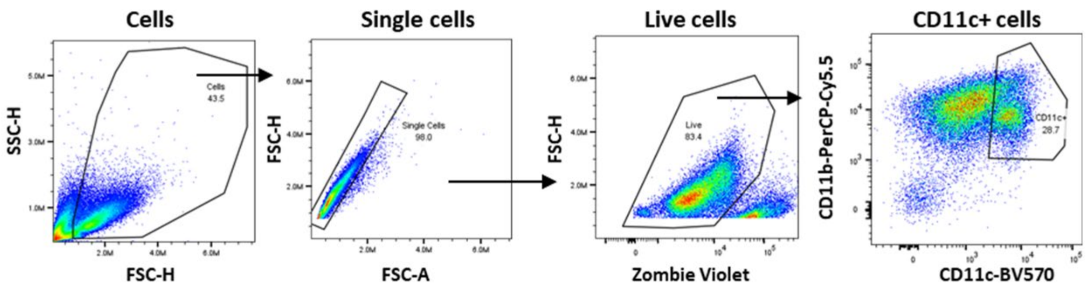

Supplemental Figure 10: Phenotyping of bone marrow-derived dendritic cells generated in the presence of adipocyte-conditioned medium. Bone marrow progenitors from C57BL/6 male mice were differentiated for 6 days in a medium supplemented with adipocyte-conditioned supernatants from ArWT or ArKO adipocytes previously incubated with testosterone or vehicle. Gating strategy to determine the proportion of CD11c^{+} cells by flow cytometry at day 6 is shown. The same gating strategy was used on day 7, after stimulation with TNFα for 24 hours, to measure the expression levels of activation molecules.

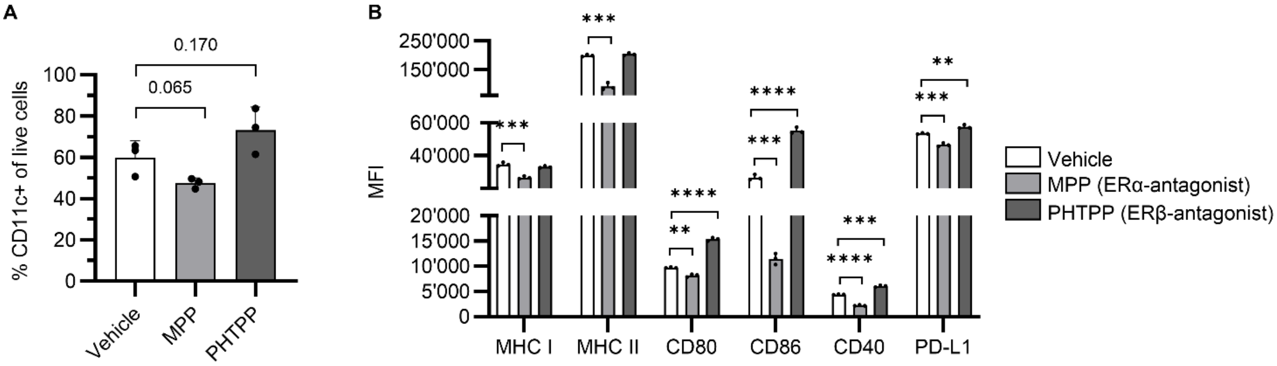

Supplemental Figure 11: ERα signaling is required for the generation of functional bone marrow-derived dendritic cells. Bone marrow progenitors from C57BL/6 male mice were differentiated for 6 days in a regular medium supplemented with estrogen receptor (ER) antagonists (MPP, PHTPP). (**A**) The percentage of CD11c^{+} cells was measured by flow cytometry at day 6. Data represent the average of three independent experiments. (**B**) Day 6 immature BMDC were stimulated with TNFα for 24 hours and the expression of activation markers was analyzed by flow cytometry at day 7. Data are representative of three independent experiments. (**A-B**) p-values from unpaired two-tailed Student's t-test are shown. $^{**}p<0.01$, $^{***}p<0.001$, $^{****}p<0.0001$. Data are depicted as mean ± SD.

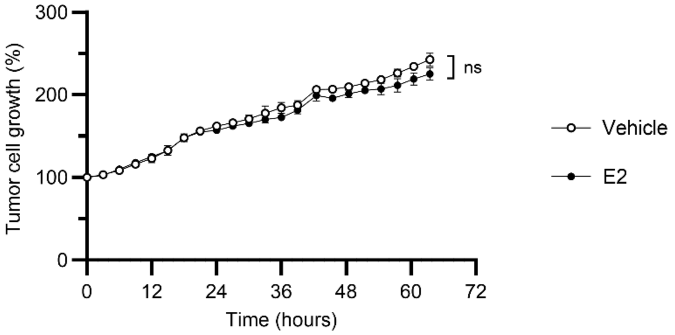

Supplemental Figure 12: Estrogens do not impact the growth of tumor cells in vitro. GFP$^+$ Renca cells were cultured in a hormone-free medium supplemented with 17β-estradiol (E2, 1 nM) and cell growth was monitored by live cell imaging over 2.5 days. Two-way ANOVA p-value was not significant: $p>0.05$. Data are depicted as mean ± SD.

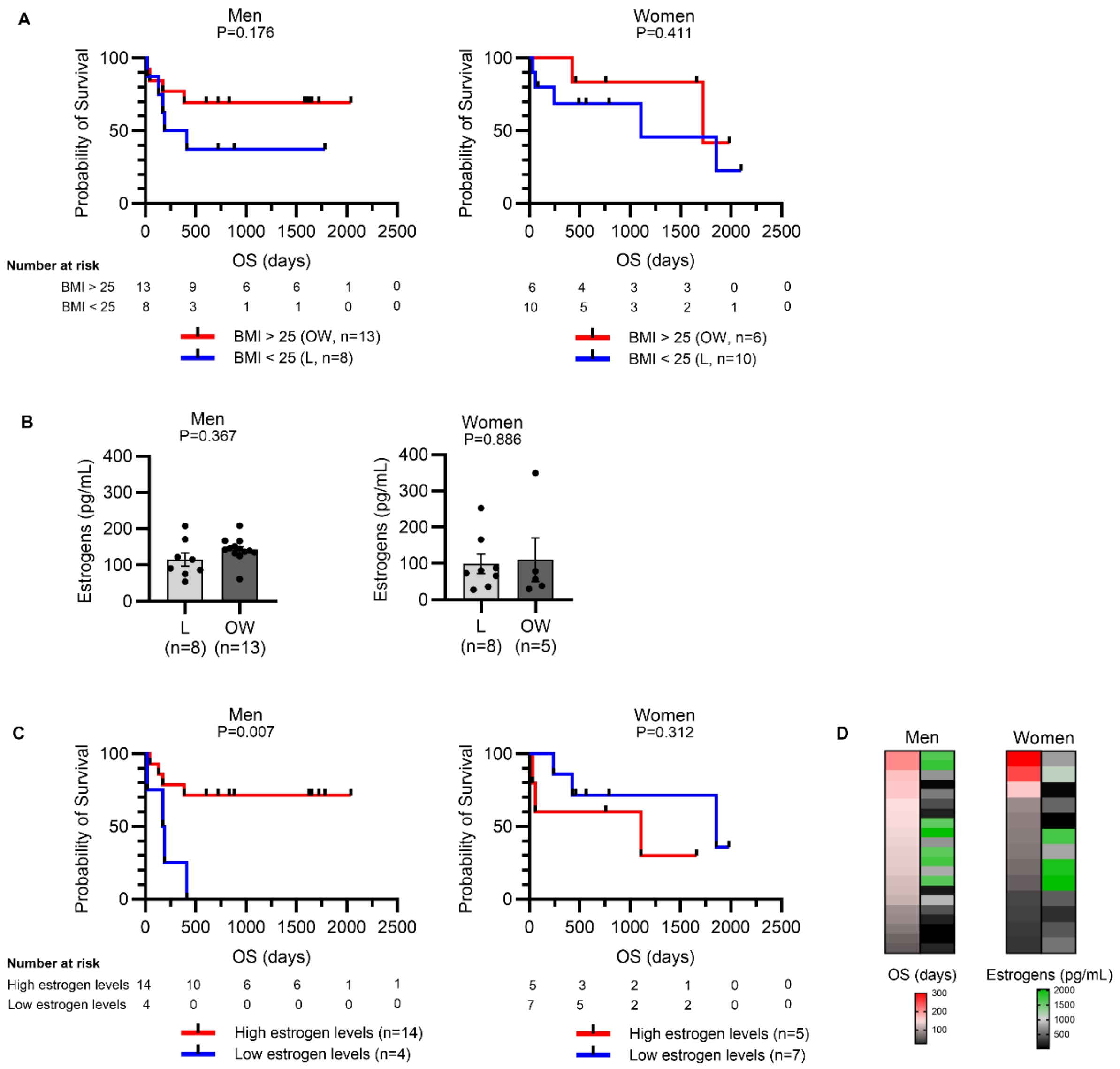

Supplemental Figure 13: Relationships between BMI, estrogen levels, and overall survival in melanoma patients receiving immune checkpoint inhibitors. (**A**) Overall survival (OS) of melanoma patients treated with ICI and stratified by BMI as lean (L; BMI < 25 kg/m^2) or overweight/obese (OW; BMI ≥ 25 kg/m^2) patients. Mantel-Cox test p-values are shown. (**B**) Levels of estrogens measured in the serum of lean or overweight/obese patients. Unpaired two-tailed Student's t-test p-values are shown. (**C**) Overall survival (OS) of patients with high versus low levels of estrogens in patients who received immune checkpoint inhibitors as first-line treatment only. Levels of estrogens were categorized as high or low compared to the median concentration of estrogens in lean men (left) or lean women (right). Mantel-Cox test p-values are shown. (**D**) Heatmap representing the level of estrogens and the OS of melanoma patients.

Supplemental tables

Supplemental Table 1: Characteristics of melanoma patients at baseline (before immune checkpoint inhibitor administration).

	Men, n (%)	Women, n (%)
**Number (%)**	21 (56.8%)	16 (43.2%)
**Age (years)**		
Median (range)	70 (40-87)	69.5 (44-87)
Elderly (>70)	10 (47.6%)	8 (50.0%)
**BMI (kg/m^2)**		
Median (range)	25.6 (22.4-30.9)	22.8 (17-48.1)
Underweight (BMI < 18.5 kg/m^2)	0 (0.0%)	2 (12.5%)
Normal weight (18.5 ≤ BMI ≤ 24.9 kg/m^2)	8 (38.1%)	8 (50.0%)
Overweight (25 ≤ BMI ≤ 29.9 kg/m^2)	12 (57.1%)	1 (6.3%)
Obese (BMI ≥ 30 kg/m^2)	1 (4.8%)	5 (31.3%)
**Subtype**		
Skin	16 (76.2%)	8 (50.0%)
Mucosal	1 (4.8%)	3 (18.8%)
Acral	1 (4.8%)	0 (0.0%)
Other	3 (14.3%)	5 (31.3%)
**Stage**		
I	4 (19%)	0 (0.0%)
II	1 (4.8%)	4 (25.0%)
III	6 (28.6%)	4 (25.0%)
IV	9 (42.9%)	7 (43.8%)
Unknown	1 (4.8%)	1 (6.3%)
**Type of immune checkpoint inhibitor**		
Ipilimumab	0 (0.0%)	1 (6.3%)
Ipilimumab + Nivolumab	9 (42.9%)	5 (31.3%)
Nivolumab	1 (4.8%)	1 (6.3%)
Pembrolizumab	11 (52.4%)	9 (56.3%)
**Treatment line**		
1	18 (85.7%)	13 (81.3%)
2	3 (14.3%)	2 (12.5%)
3	0 (0.0%)	1 (6.3%)
**PD-L1 expression**		
< 1%	6 (28.6%)	2 (12.5%)
1-49%	8 (38.1%)	9 (56.3%)
> 50%	0 (0.0%)	0 (0.0%)
Unknown	7 (33.3%)	5 (31.3%)

BMI, body mass index; PD-L1, programmed death-ligand 1.
